# Baishaoluoshi Decoction Mitigates Post‐Stroke Spasticity by Targeting Synaptic Plasticity Through the Nogo‐A/NgR Signaling Pathway

**DOI:** 10.1002/brb3.71170

**Published:** 2025-12-31

**Authors:** Xiongxing Sun, Shanshan Zeng, Shigao Lin, Lingying Wu, Xukun Tang, Jiajian Zhu, Yuhui Zhang, Lu Li, Ziming Chen, Xinyu Deng, Dahua Wu, Le Xie

**Affiliations:** ^1^ Graduate School of Hunan University of Chinese Medicine Changsha Hunan Province China; ^2^ Hunan Provincial Hospital of Integrated Traditional Chinese and Western Medicine (The Affiliated Hospital of Hunan Academy of Traditional Chinese Medicine) Changsha Hunan Province China

**Keywords:** Baishaoluoshi Decoction, Nogo receptor, post‐stroke spasticity, synaptic plasticity

## Abstract

**Objective:**

This study systematically investigated the effects and molecular mechanisms of Baishaoluoshi Decoction (BD) on synaptic plasticity in rats with post‐stroke spasticity (PSS).

**Methods:**

A rat model of PSS was established using middle cerebral artery occlusion. Behavioral assessments, including modified neurological severity scores, rotarod tests, and the Modified Ashworth Scale, were employed. Synaptic ultrastructure was analyzed using transmission electron microscopy (TEM). The molecular mechanisms were explored using immunofluorescence and western blot analyses to evaluate protein expression (Nogo‐A, Nogo receptor (NgR), RhoA, Collapsin Response Mediator Protein 2 (CRMP2), and AGG) and NgR/Olig2 colocalization. BD was also tested in combination with the NgR antagonist NEP1‐40.

**Results:**

BD significantly ameliorated the neurological deficits, prolonged rotarod fall latency, and reduced spasticity. TEM revealed that BD restored the synaptic ultrastructure in the peri‐infarct regions by increasing postsynaptic density (PSD) thickness and length, and narrowing synaptic clefts. BD downregulates synaptic‐plasticity‐related proteins (Nogo‐A, NgR, RhoA, AGG, and CRMP2) and attenuates oligodendrocyte‐mediated inhibitory signaling via reduced NgR/Olig2 co‐localization. BD combined with NEP1‐40 exhibits synergistic therapeutic efficacy.

**Conclusion:**

BD alleviates PSS by enhancing synaptic plasticity and suppressing inhibitory signaling through multitarget modulation of neuron–glia interactions. These findings highlight BD as a promising therapeutic intervention for PSS, which is supported by molecular evidence of its effects on synaptic remodeling and functional recovery.

## Graphical abstract

Baishaoluoshi Decoction alleviates post‐stroke spasticity by downregulating inhibitory proteins (Nogo‐A/NgR) in the peri‐infarct brain region and reducing NgR/Olig2 co‐localization in oligodendrocytes, thereby enhancing synaptic plasticity.

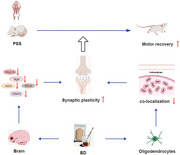

## 1 Introduction

Post‐stroke spasticity (PSS), a common motor and sensory disorder that occurs at various stages after a stroke, represents a major clinical challenge in neurorehabilitation (Chen et al. 2025). As the most prevalent type of cerebrovascular accident, cerebral infarction induces upper motor neuron damage, characterized by intermittent or persistent increases in hypertonic stretch reflexes and tendon hyperreflexia (Bavikatte et al. 2021). Spastic paralysis is a primary motor dysfunction in patients with stroke and a leading cause of severe disability. The associated pain, muscle rigidity, limb deformities, and mobility limitations necessitate long‐term care and intervention, imposing significant economic and psychological burdens on patients and their caregivers.

Despite decades of research, the precise pathogenesis of PSS remains unclear. Emerging evidence has implicated the Nogo‐A/Nogo receptor (NgR) signaling axis as a critical regulator of post‐ischemic synaptic plasticity and axonal regeneration (Tang 2020). Following neural injury, oligodendrocytes markedly upregulate the expression level of Nogo‐A, which undergoes proteolytic processing into bioactive fragments (Nheu et al. 2022). These fragments are predominantly secreted via exosomes and exert inhibitory effects through the following distinct mechanisms: (1) RhoA/ROCK‐mediated growth cone collapse via myosin II activation (Wang et al. 2020); (2) collapsin response mediator protein 2 (CRMP2)‐dependent suppression of microtubule elongation through NgR1/PlexinA2 complex formation (Sekine et al. 2019); and (3) AGG‐induced chemical barrier formation via PTPσ‐dependent blockade of autophagosome‐lysosome fusion (Kurihara et al. 2017). Collectively, these mechanisms severely constrain synaptic plasticity and functional recovery in patients with PSS.

Currently, the clinical management of PSS focuses on alleviating spasticity and improving quality of life, primarily through oral medications (e.g., baclofen, tizanidine), intramuscular botulinum toxin A injections, and rehabilitation therapies. However, long‐term clinical observations indicate that, while Western pharmacological interventions provide symptomatic relief, depressant effects on the central nervous system may impede functional recovery. In contrast, growing evidence has highlighted the potential benefits of traditional Chinese medicine in ameliorating PSS. Our previous studies revealed that Baishaoluoshi Decoction (BD) significantly improves neurological deficits and limb spasticity in rats with PSS, demonstrating its multi‐target synergistic antispastic effects (Xie et al. 2021; Zeng et al. 2022; Le et al. 2023). To further elucidate its therapeutic mechanisms, we established a rat model of PSS via middle cerebral artery occlusion (MCAO) to decipher the molecular actions of BD on synaptic plasticity in PSS.

## 2 Materials and Methods

### 2.1 Animals

Healthy 8‐week‐old male Sprague–Dawley rats weighing 250–300 g were provided by Hunan SJA Laboratory Animal Co., Ltd. (Changsha, China; No. 430727251100338784). All rats were housed in individually ventilated cages at a temperature of 22 ± 1°C and 60% relative humidity, with 12 h of alternating light and darkness. Food and water were provided. The use of rats and their treatments were approved by the Laboratory Animal Welfare and Ethics Committee of the Hunan Academy of Chinese Medicine (Changsha, China; No. SY2024‐0017), and all experiments were performed in strict compliance with the relevant regulations.

### 2.2 Crude Drugs

All crude drugs were purchased from Changsha Xinlin Pharmaceutical Co., Ltd. (Changsha, China) and the samples were authenticated by Prof. Jinsong Wu (Hunan Provincial Hospital of Integrated Traditional Chinese and Western Medicine, The Affiliated Hospital of Hunan Academy of Chinese Medicine, Changsha, China).

### 2.3 Experimental Methods

#### 2.3.1 BD Preparation

BD was prepared by combining its primary herbal constituents, baishao (*Paeoniae radix Alba*) and luoshiteng (*Trachelospermum jasminoides*) in a 1:1 ratio. Formula granules of baishao (No. 16050101) and luoshiteng (No. 16082053) were procured as standardized extracts from Changsha Xinlin Pharmaceutical Co. Ltd. (Changsha, China).

#### 2.3.2 Quality Analysis of BD Using High‐Performance Liquid Chromatography

The chromatographic analysis of BD was performed using agilent 1290 high‐performance liquid chromatography (HPLC) system (Agilent Technologies Inc., Santa Clara, USA). An InertSustain AQ‐C18 column (4.6 × 250 mm, 5 µm,) was employed with mobile phase A as methanol (TEDIA Co., Ltd., USA, No. 24116690) and mobile phase B as 0.1% formic acid solution (Tianjin Kemiou Chemical Reagent Co., Ltd., Tianjin, China, No. 20220404) for gradient elution. The flow rate was 1.0 mL/min, the column temperature was maintained at 30°C, and the detection wavelength was set to 230 nm. The authenticated reference standard, paeoniflorin (No. 110736–202246) was sourced from National Institutes for Food and Drug Control (China), and tracheloside (No. CHB250107) and trachelogenin (No. CHB250310) were sourced from Chengdu Clooma Biotechnology Co., Ltd. (China).

#### 2.3.3 Establishment of Rat Model of PSS

Intraluminal MCAO was used to establish a rat model of cerebral ischemia, as previously described (Zhu et al. 2015). Briefly, the rats were anesthetized with 2% pentobarbital sodium (0.23 mL/100 g) through intraperitoneal injection after fasting for 4–6 h. A heating pad was used to maintain the rats’ body temperature throughout the process. After disinfecting the skin, the right common carotid artery (CCA), internal carotid artery (ICA), and external carotid artery (ECA) were exposed. The ECA was ligated using a 4‐0 surgical suture, and the ICA was temporarily clamped using a microarterial clip. Subsequently, the proximal and distal ends of the CCA were ligated. The right middle cerebral artery (MCA) was occluded with an MCAO monofilament (Beijing Cinontech Biotech Co. Ltd., Beijing, China; 0.26 mm in diameter) by inserting it from the right CCA and advancing into the ICA until it blocked the origin of the MCA. Finally, the monofilament was trimmed and the neck skin was disinfected and sutured. The sham group underwent the same surgical procedure except for insertion of the monofilament.

#### 2.3.4 Animal Grouping and Treatment

**FIGURE 1:**
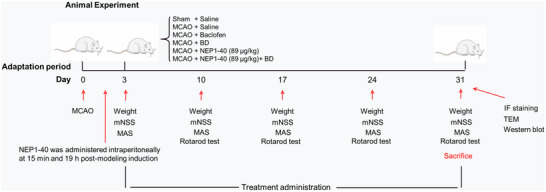
Animal experiment flowchart.

The rats were divided into the following six groups using the random number table method: sham‐operated group (S), model group (M), BD group, baclofen group (B) (positive reference drug), antagonist group, and BD+ antagonist group, with 12 rats per group. At 15 min and 19 h after the successful establishment of the model, both the antagonist group and the BD+ antagonist group were intraperitoneally injected with the NgR antagonist NEP1‐40 (Medchemexpress LLC, Shanghai, China, No. 150964) at a dose of 89 µg/kg 97.5% phosphate‐buffered saline (Wuhan Pinuofei Biological Technology Co., Ltd., Wuhan, China, NO. PN0031) and 2.5% dimethyl sulfoxide (Macklin Biochemical Co., Ltd., Shanghai, China, No. D6258). Rats in the BD and BD+ antagonist groups were intragastrically administered 5.4 g/kg/d of the crude drug, according to a previous study (Le et al. 2023). Group B was intragastrically administered 5.4 mg/kg/d baclofen (Fuan Pharmaceutical Group Co., Ltd., Ningbo, China, CAS: 1134‐47‐0). Each group was intragastrically administered the corresponding drugs at a volume of 10 mL/kg of body weight, whereas rats in the S, M, and antagonist groups were administered an equal volume of distilled water. The drug/water was administered once a day, for 4 consecutive weeks from the third day after surgery (Figure 1).

#### 2.3.5 Modified Neurological Severity Score

Neurological deficits were assessed using the modified neurological severity score (mNSS), which is a composite metric for evaluating motor, sensory, and reflex functions (Table 1). One point was assigned for failure to perform a task or the absence of a specific reflex, with higher total scores indicating more severe neurological impairment (Pan et al. 2020).

**TABLE 1:** Modified neurological severity score.

Test item	Score
Motor function	
Raising the rat by the tail:	
Flexion of forelimb or hindlimb	1
Head moved more than 10°C to the vertical axis within 30 s	1
Walking on the ground	
Normal walking	0
Circling toward the paretic side	1
Fall down to the paretic side	2
Abnormal movement	
Immobility, staring, tremor and pilo‐erection	1
Myodystony and irritability	1
Sensory function	
Ipsilateral forelimb retracts after acupuncture	0
Ipsilateral forelimb does not retract after acupuncture	1
Reflex deficiency	
Pinna reflex (head shake when touching the external auditory meatus)	1
Corneal reflex (blink when lightly touching the cornea with cotton)	1
Startle reflex (Motor response to sudden appearance of the noise)	1
Total score	11

#### 2.3.6 Rotarod Test

The rotarod test (Beijing Zhongshi Dichuang Technology Development Co., Ltd., Beijing, China, ZS‐RDM‐DS) was used to assess motor and coordination functions in rats. Each test cycle was completed over 2 days. On the first day (training session), the animals were placed on the rotating rod at a speed of 20 rpm for 2 min to familiarize themselves with the environment. On the second day (formal test), the animals were required to crawl on a gradually accelerating rotating rod to evaluate their balance coordination and muscle endurance. The test ended when a rat fell off the rod, and a second trial was conducted after a 10‐min rest period, with three trials in total. The mean fall latency (s), fall speed (m/s), and rotation distance (m) were recorded. The specific parameters included acceleration from the initial speed to 5 rpm within 5 s, followed by a uniform acceleration to 20 rpm over 40 s, maintenance at 20 rpm for 5 s, acceleration to 40 rpm over 40 s, and final continuation at 40 rpm until the termination of the test.

#### 2.3.7 Spasticity Evaluation

Spasticity in the hemiplegic limb was assessed using the Modified Ashworth Scale (MAS). This scoring system defines six grades: grade 0, no detectable increase in muscle tone; grade 1, slight resistance at the end of passive movement; grade 1+, mild resistance during less than half of the passive range; grade 2, resistance throughout most of the range; grade 3, severe resistance across the entire range accompanied by dyskinetic movements; and grade 4, complete rigidity with the limb fixed in flexion or extension (Bohannon and Smith 1987). The MAS scores (0, 1, 1+, 2, 3, and 4) were assigned numerical values (0, 1, 2, 3, 4, and 5, respectively) (Li et al. 2020).

#### 2.3.8 Ultrastructural Observations Using Transmission Electron Microscopy

To examine the synaptic ultrastructure via transmission electron microscopy (TEM), peri‐infarct brain tissue was sectioned into 1–3 mm cubic fragments and sequentially fixed in electron microscopy fixative (Wuhan Pinuofei Biological Technology Co., Ltd., Wuhan, China, NO. PN0019) and then 1% osmium tetroxide (Ted Pella, Inc., Redding, USA, No. 18456) for 2 h. Following dehydration in a graded ethanol series and epoxy resin embedding, ultrathin sections with a thickness of 60–80 nm were prepared and subjected to double staining with uranyl acetate and lead citrate for 15 min. Synaptic morphology, particularly gray type I synapses, was analyzed using a Hitachi TEM system (Hitachi, Tokyo, Japan, HT7800) with Image‐Pro Plus 6.0.

#### 2.3.9 Immunofluorescent Staining Detection of NogoA, NgR, RhoA, AGG, and CRMP2 Proteins

Rat brain tissues were fixed in 4% paraformaldehyde, embedded in paraffin, and sectioned at 4–6 µm. The sections were deparaffinized in xylene (Sinopharm Chemical Reagent Co., Ltd., Shanghai, China, No. 10023418), rehydrated using a graded ethanol series, and subjected to microwave‐assisted antigen retrieval in citrate buffer (pH 6.0). After blocking with goat serum (Wuhan Pinuofei Biological Technology Co., Ltd., Wuhan, China, NO. PN0038), the sections were incubated with primary antibodies (against Nogo‐A (1:100, Affinity, Beijing, China, No. DF8581), NgR (1:100, Affinity, Beijing, China, No. DF13593), RhoA (1:100, Affinity, Beijing, China, No. AF6352), AGG (1:100, Affinity, Beijing, China, No. DF7561), or CRMP2 (1:100, Affinity, Beijing, China, No. AF6459)) overnight at 4°C. Secondary antibodies and DAPI were applied sequentially, followed by mounting in antifade mounting medium (Wuhan Pinuofei Biological Technology Co., Ltd., Wuhan, China, NO. PN0024). Fluorescence signals were imaged and analyzed using Image‐Pro Plus 6.0. Pearson's correlation, Pearson–Spearman overlap, and Mander's colocalization coefficients were used to quantify the proteins’ colocalization (Dunn et al. 2011; Adler and Parmryd 2013).

#### 2.3.10 Western Blotting Detection of NogoA, NgR, RhoA, AGG, and CRMP2 Proteins

The rats were euthanized, and brain tissues were promptly collected. Total protein was extracted using radioimmunoprecipitation assay lysis buffer (Kangwei Century Biotechnology Co., Ltd., Taizhou, China, No. CW2333S), and concentrations were determined using a bicinchoninic acid assay. Proteins were resolved on 10% sodium dodecyl sulfate‐polyacrylamide gel electrophoresis and transferred onto polyvinylidene fluoride membranes. The membranes were incubated overnight at 4°C with primary antibodies (anti‐β‐actin (1:100, Affinity, Beijing, China, No. AF7018), ‐Nogo‐A, ‐NgR, ‐RhoA, ‐AGG, and ‐CRMP2 antibodies), followed by species‐matched secondary antibodies. Protein bands were visualized using enhanced chemiluminescence and quantified using Image‐Pro Plus 6.0. Uncropped blot images for all replicates are provided in the Supplementary Material.

### 2.4 Ethics Approval and Consent to Participate

The present study was approved by the Laboratory Animal Welfare and Ethics Committee, Hunan Academy of Chinese Medicine (Changsha, China).

### 2.5 Statistical Analysis

SPSS Statistics for Windows, version 20.0 (IBM, Armonk, NY, USA), was used for data analysis. The results are expressed as the mean ± standard deviation. One‐way analysis of variance was used to test the normalization and homogeneity of variance. Because the variance was small, a pairwise comparison was conducted using the significance method. When the variance was not uniform, the Games–Howell method was employed. A *p*‐value < 0.05 was considered statistically significant.

## 3 Results

### 3.1 HPLC Chromatogram of BD

The HPLC fingerprint chromatogram of BD revealed that peak 1 (paeoniflorin) was derived from baishao, whereas peaks 2 (tracheloside) and 3 (trachelogenin) originated from luoshiteng (Figure 2A). Chromatograms of the three batches of BD were established and showed a high degree of similarity, indicating that BD from the different batches was stable and consistent (Figure 2B). Based on the HPLC fingerprint analysis, a precise and stable quality control method for BD was established.

FIGURE 2High‐performance liquid chromatography chromatogram of Baishaoluoshi Decoction. **(A)** Reference sample of paeoniflorin, tracheloside, and trachelogenin, the main component of BD (Baishao and Luoshiteng at a ratio of 1∶1). Peaks 1–3 represented paeoniflorin, tracheloside and trachelogenin. (**B**) Chromatograms of the three batches of BD were established and showed a high degree of similarity.

**Figure:**
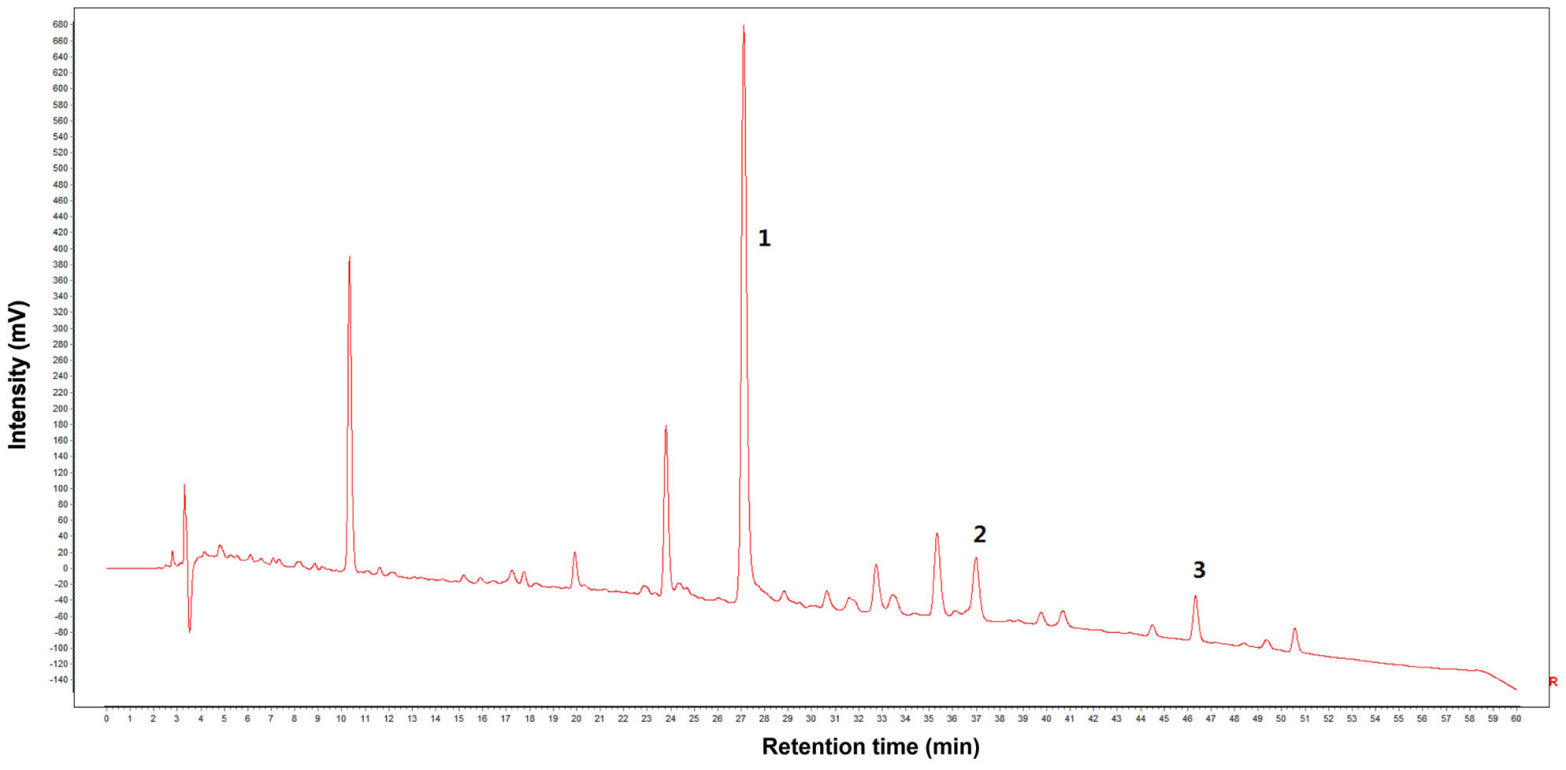

**Figure:**
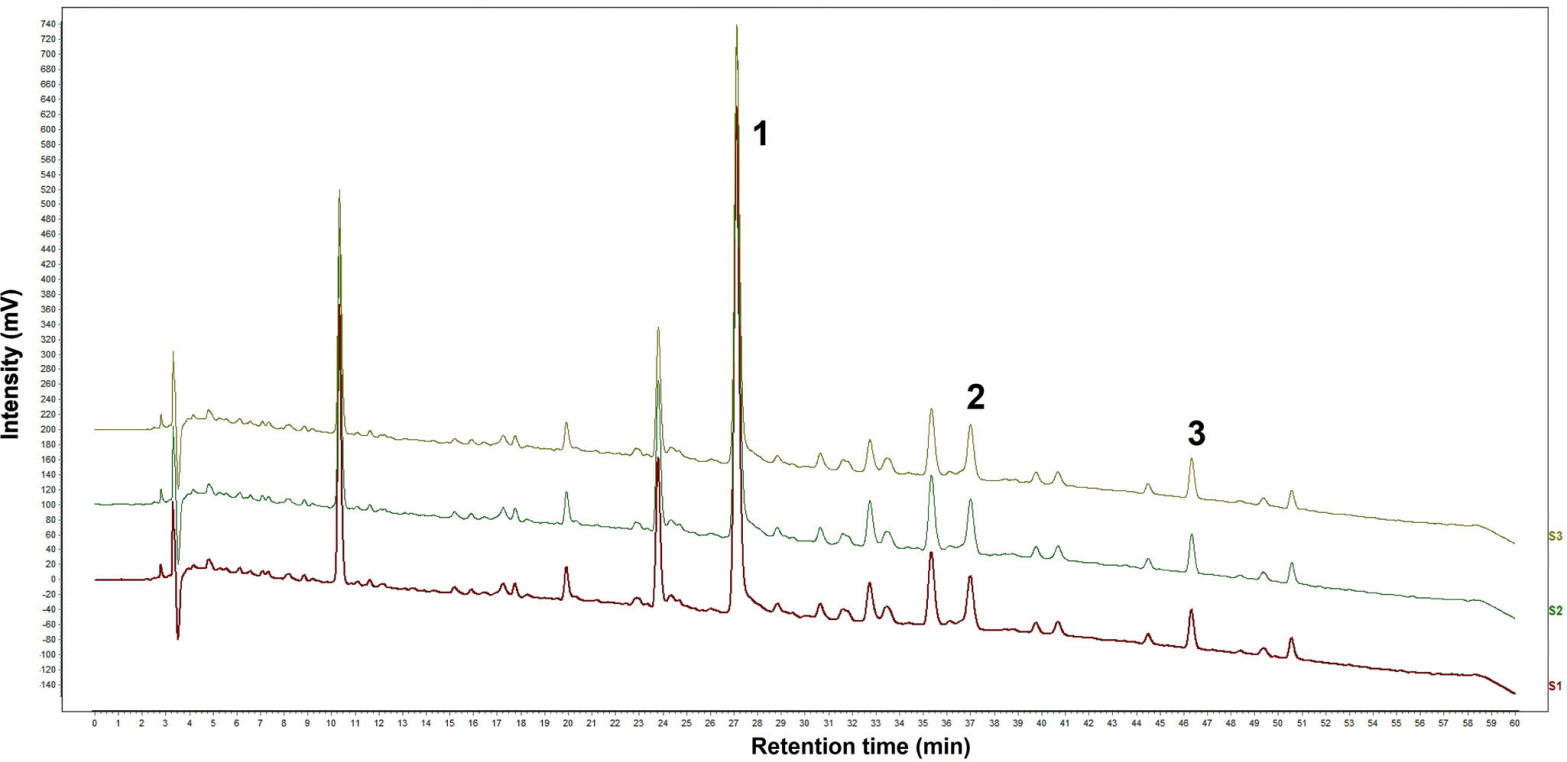

### 3.2 Neuroprotective Effects of BD in Rats With PSS

**FIGURE 3:**
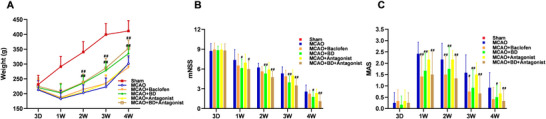
Neuroprotective effects of BD in rats with PSS. (**A**) Comparison of weights in each group at different time points. The weights of rats in each group were measured at different times, 3 days after the model establishment and 1, 2, 3, and 4 weeks after the intervention. (**B**) Comparison of the mNSS in each group at different time points. The mNSS was used to evaluate neurological deficits 3 days after model establishment and 1, 2, 3, and 4 weeks after the intervention. (**C**) Comparison of the MAS in each group at different time points. The MAS was used to evaluate the spasticity of the affected forelimb 3 days after model establishment and 1, 2, 3, and 4 weeks after the intervention. The PSS model was established using MCAO, and the intervention began 3 days after the model was established and continued for 4 weeks. Rats were treated daily with BD (5.4 g/kg in MCAO + BD, MCAO + BD+ antagonist), baclofen (5.4 mg/kg), or normal saline (MCAO, Sham, MCAO + antagonist) by gavage for 4 weeks. BD, Baishaoluoshi Decoction; PSS, post‐stroke spasticity; mNSS, modified neurological deficit score; MAS, Modified Ashworth Score; MCAO, middle cerebral artery occlusion. ^##^ *p* < 0.01 and ^#^ *p* < 0.05, compared with the model group (*n* = 12).

Approximately 10 days after MCAO surgery, the rats exhibited significant weight loss, which broadly reflected their health status and indicated severe ischemic stroke injury (Figure 3A). The BD and BD+ antagonist groups showed faster weight recovery and growth rates than the baclofen and model groups (*p* < 0.01), suggesting that BD improved the survival of rats with PSS. Compared with the model group, the BD and BD+ antagonist groups showed significant amelioration of the mNSS at weeks 2 and 3 (*p* < 0.01), with additional effects observed 1 and 4 weeks post‐intervention (*p* < 0.05; Figure 3B).

### 3.3 Spasticity in Rats With PSS Was Ameliorated by Treatment With BD

The MASs indicated that the positive control drug baclofen significantly alleviated spasticity in the hemiplegic limbs of rats with PSS. BD also mitigated spasticity in rats with PSS at 1, 2, 3, and 4 weeks post‐intervention (*p* < 0.01). However, combined BD and antagonist treatment demonstrated superior efficacy at reducing spasticity compared to BD alone at all measured time points (1, 2, 3, and 4 weeks post‐intervention, *p* < 0.01; Figure 3C)

### 3.4 BD Enhanced Motor Function of Rats With PSS

**FIGURE 4:**
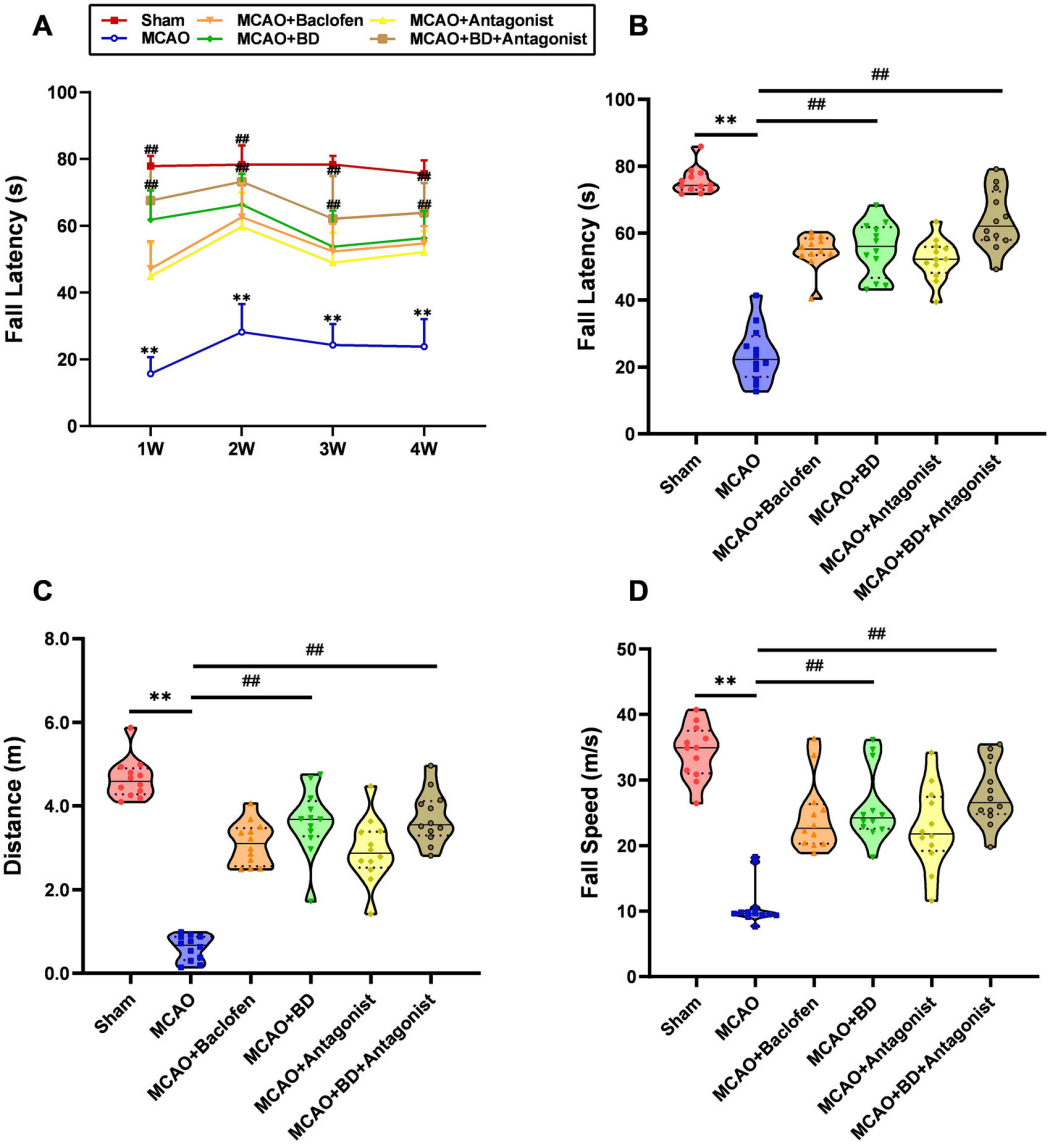
BD enhanced motor function in rats with PSS. (**A**) Comparison of fall latencies in each group at different time points. The fall latency of rats in each group was detected at the following time points: 3 days after model establishment and 1, 2, 3, and 4 weeks after the intervention. (**B**) Comparison of fall latency in each group 4 weeks after the intervention. (**C**) Comparison of the distance traveled for each group 4 weeks after intervention. (**D**) Comparison of fall speed in each group 4 weeks after the intervention. **p < 0.01 compared to the sham group, ^##^ *p* < 0.01 compared to the model group (*n* = 12).

We compared motor coordination and muscle fatigue endurance in rats by recording their latency to fall off a rotarod. At the fourth week post‐intervention, compared to the sham group, the model group exhibited a significant decrease in the latency to fall (*p* < 0.01; Figure 4B), reduced total crawling distance (*p* < 0.01; Figure 4C), and slower falling speed (*p* < 0.01; Figure 4D). In contrast, both the BD and BD+ antagonist groups showed significantly increased latency to fall (*p* < 0.01), enhanced total crawling distance (*p* < 0.01), and accelerated falling speed (*p* < 0.01). The BD+ antagonist treatment showed stronger therapeutic efficacy than BD treatment alone. Additionally, at 1, 2, 3, and 4 weeks post‐intervention, the model group displayed a significantly shorter latency to fall than the sham group (*p* < 0.01), while the BD and BD+ antagonist groups showed markedly prolonged latency (*p* < 0.01; Figure 4A). Notably, the BD+ antagonist treatment outperformed BD treatment alone in terms of efficacy, indicating that BD enhanced long‐term motor capacity, improved motor coordination, and increased muscle endurance in rats with PSS rats.

### 3.5 BD Rescued Synaptic Structure in Rats With PSS

**FIGURE 5:**
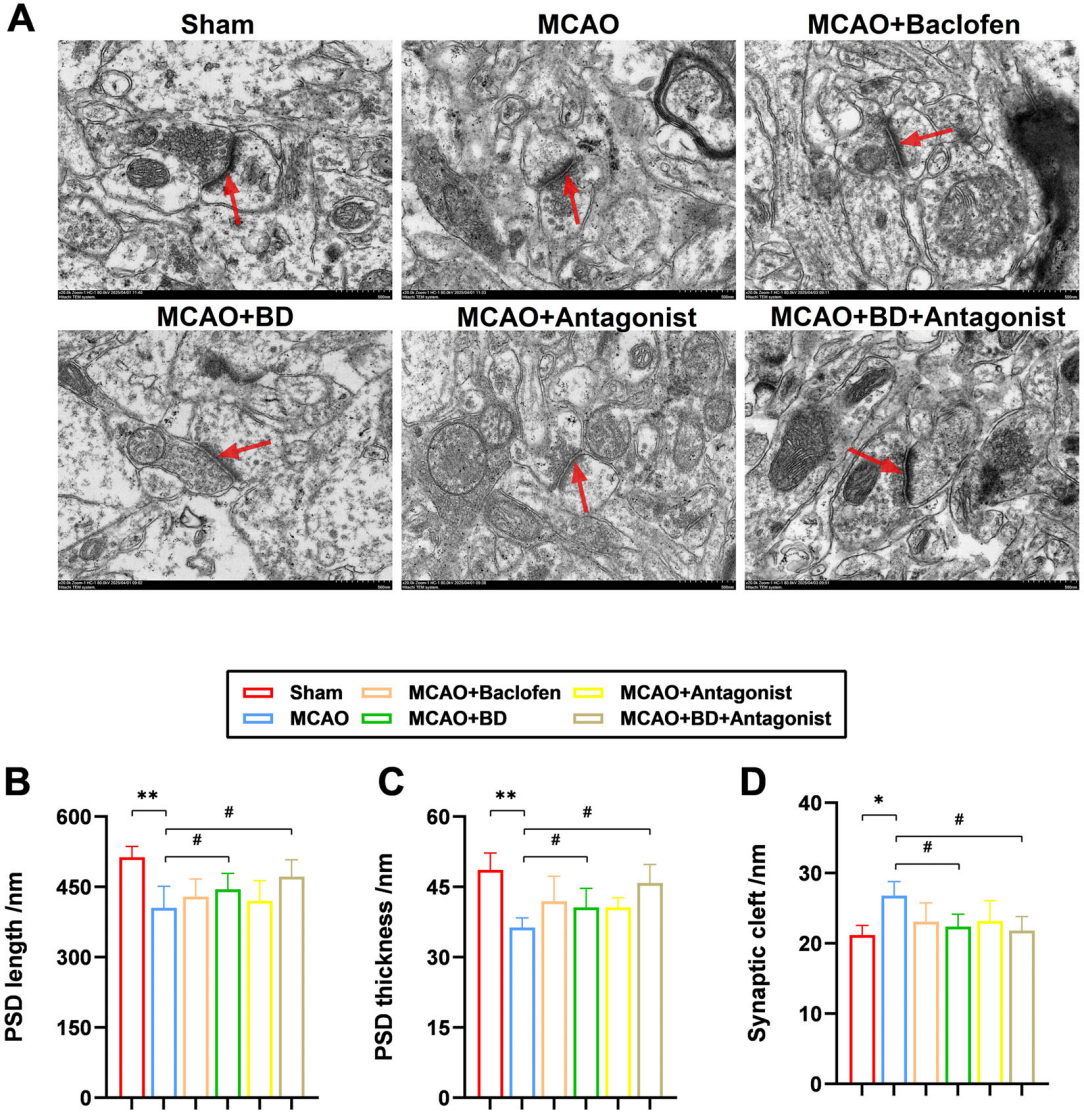
Effects of BD on the synaptic structure in rats with PSS. (**A**) Synaptic structures observed using transmission electron microscopy (×20,000). The red arrows represent synapses. Comparison of (**B**) postsynaptic density (PSD) length, (**C**) the thickness of the brain tissue, and (**D**) the synaptic cleft in the brain tissue around the infarcts. **p < 0.01 and *p < 0.05 compared to the sham group, ^##^ *p* < 0.01 and ^#^ *p* < 0.05 compared to the model group (*n* = 3).

Additionally, ultrastructural changes in the synapses at the site of ischemic injury in rats with PSS were observed using TEM. In the sham group, the synapses were evenly distributed, with elongated and robust postsynaptic densities (PSDs) and narrow synaptic clefts (Figure 5A). However, in the model group, the synaptic ultrastructure appeared blurred, accompanied by alterations in the structural parameters of the synaptic interface, including reduced length and thickness of the PSD (*p* < 0.01; Figure 5B, C), and a widened synaptic cleft (*p* < 0.05; Figure 5D). Treatment with BD and BD+ antagonists significantly increased the number of damaged synapses, resulting in relatively intact synaptic structures. The synaptic cleft was also significantly narrowed in rats with PSS compared with those in the model group (*p* < 0.05). These results demonstrated that BD can repair synaptic structural damage caused by ischemic injury, with the BD+ antagonist treatment exhibiting superior therapeutic efficacy compared with BD alone.

### 3.6 BD Promoted the Expression of Synaptic‐Plasticity‐Associated Proteins in Peri‐Infarct Brain Tissue

**FIGURE 6:**
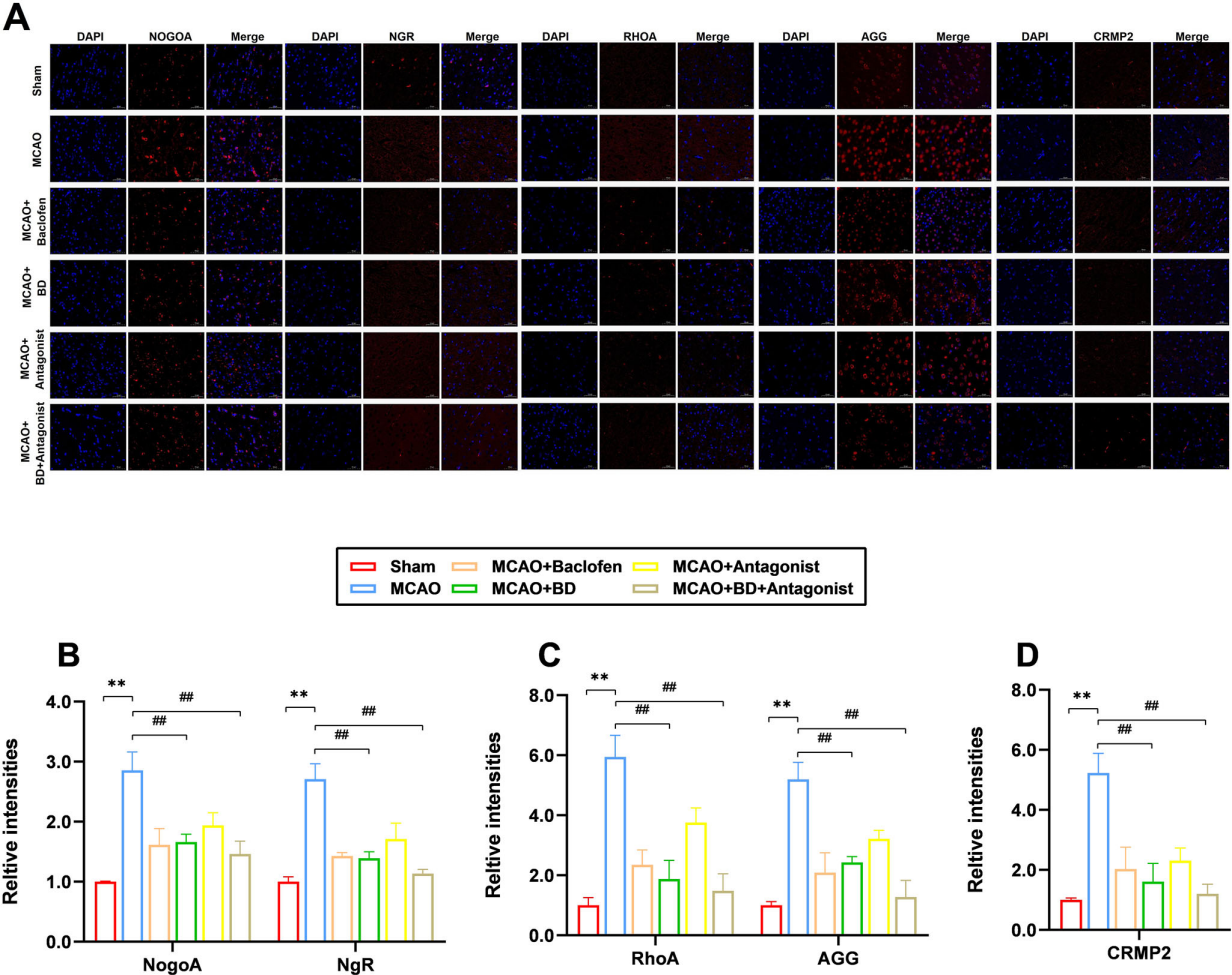
Immunofluorescence analysis of the effects of BD on synaptic‐plasticity‐associated proteins in rats with PSS. (**A**) Protein expression levels of Nogo‐A, NgR, Rhoa, AGG, and CRMP2 in the brain tissue around the infarct were detected using immunofluorescence staining, 4 weeks after the intervention. (**B**) Relative expression levels of Nogo‐A and NgR. (**C**) Relative expression levels of Rhoa and AGG. D: Relative expression levels of CRMP2. **p < 0.01 compared to the sham group, ^##^ *p* < 0.01 compared to the model group (*n* = 3).

NgR, RhoA, and CRMP2 play critical roles in synaptic development, maturation, and plasticity by modulating cytoskeletal dynamics, receptor trafficking, and signal transduction. Furthermore, Nogo‐A and its ligands influence synaptic stability through NgR, whereas extracellular matrix (ECM) components, such as AGG, participate in plasticity regulation by stabilizing the synaptic microenvironment. Immunofluorescence staining results demonstrated that treatment with BD and BD+ antagonist significantly reduced the expression levels of synaptic‐plasticity‐related proteins (Nogo‐A, NgR, RhoA, AGG, and CRMP2) compared to their levels in the model group, in the peri‐infarct brain tissue (*p* < 0.01; Figure 6). This suggested that BD promoted neural growth and enhanced synaptic plasticity, with the BD+ antagonist treatment exhibiting superior therapeutic efficacy.

In addition, western blotting results showed that compared to their levels in the model group, the expression levels of synaptic‐plasticity‐related proteins Nogo‐A, NgR, RhoA, CRMP2, and AGG in the peri‐infarct brain tissue were significantly reduced following treatment with BD and BD+ antagonist (*p* < 0.01; Figure 7). This finding supports the idea that BD promotes neural growth and improves synaptic plasticity. Notably, the expression levels of Nogo‐A, NgR, RhoA, and CRMP2 were lower in the BD+ antagonist group than the BD group. However, unlike the immunofluorescence findings, AGG expression levels were higher in the BD+ antagonist group than the BD group but were significantly lower than those of the model group (*p* < 0.01).

FIGURE 7Western blotting analysis of the effects of BD on synaptic‐plasticity‐associated proteins in rats with PSS. (**A**) Protein expression levels of Nogo‐A, NgR, Rhoa, AGG, and CRMP2 in the brain tissue around the infarct were detected using western blotting analysis, 4 weeks after the intervention. β‐actin was used as the internal control. (**B**) Relative expression levels of Nogo‐A and NgR. (**C**) Relative expression levels of Rhoa and AGG; D: Relative expression levels of CRMP2. **p < 0.01 compared to the sham group, ^##^ *p* < 0.01 compared to the model group (*n* = 3). The full, uncropped images of all Western blot replicates (three independent experiments) are provided in the Supplementary Material (Supplementary Files 1–38). The blot images presented in the figures are representative of these consistent results.

**Figure:**
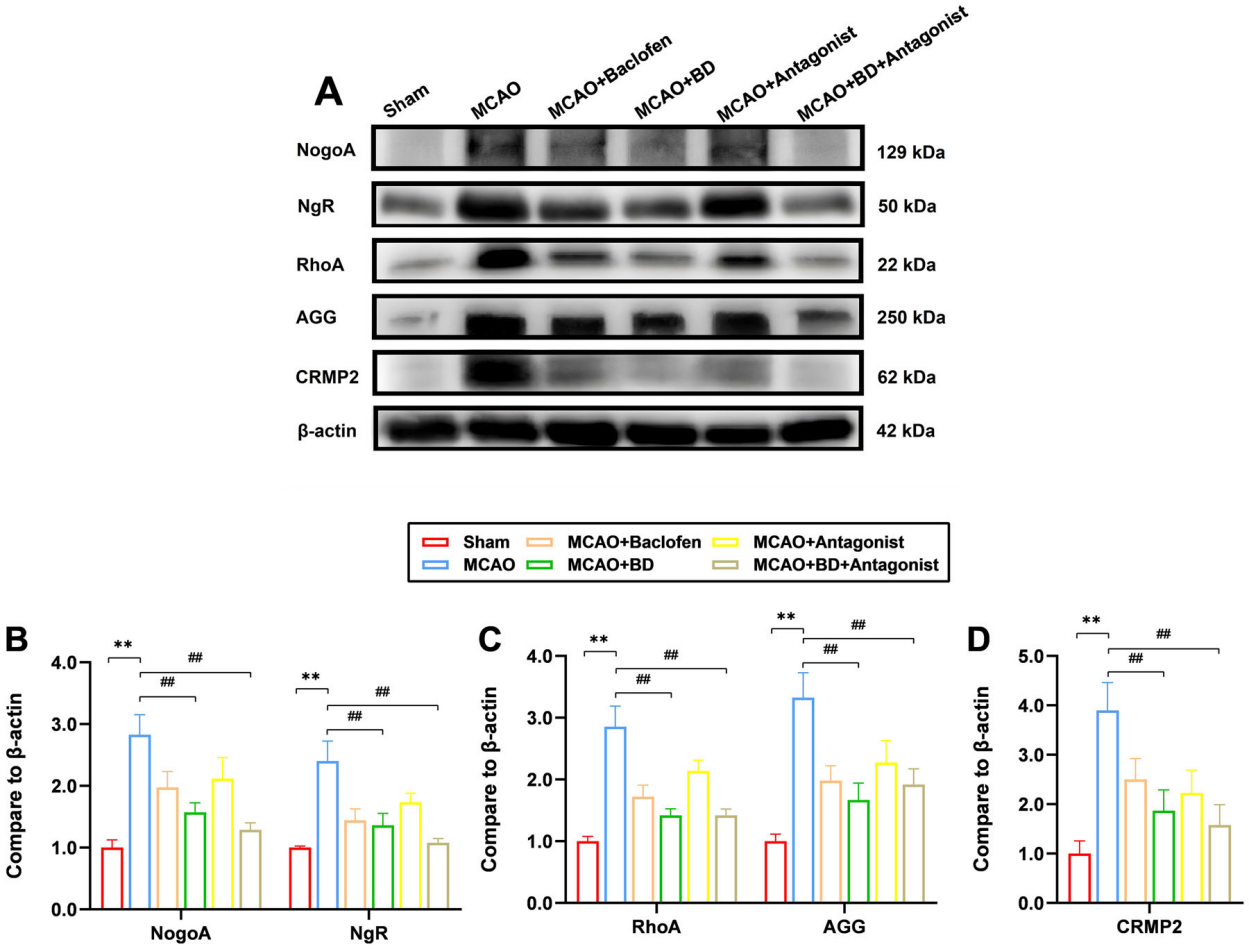

**Figure:**
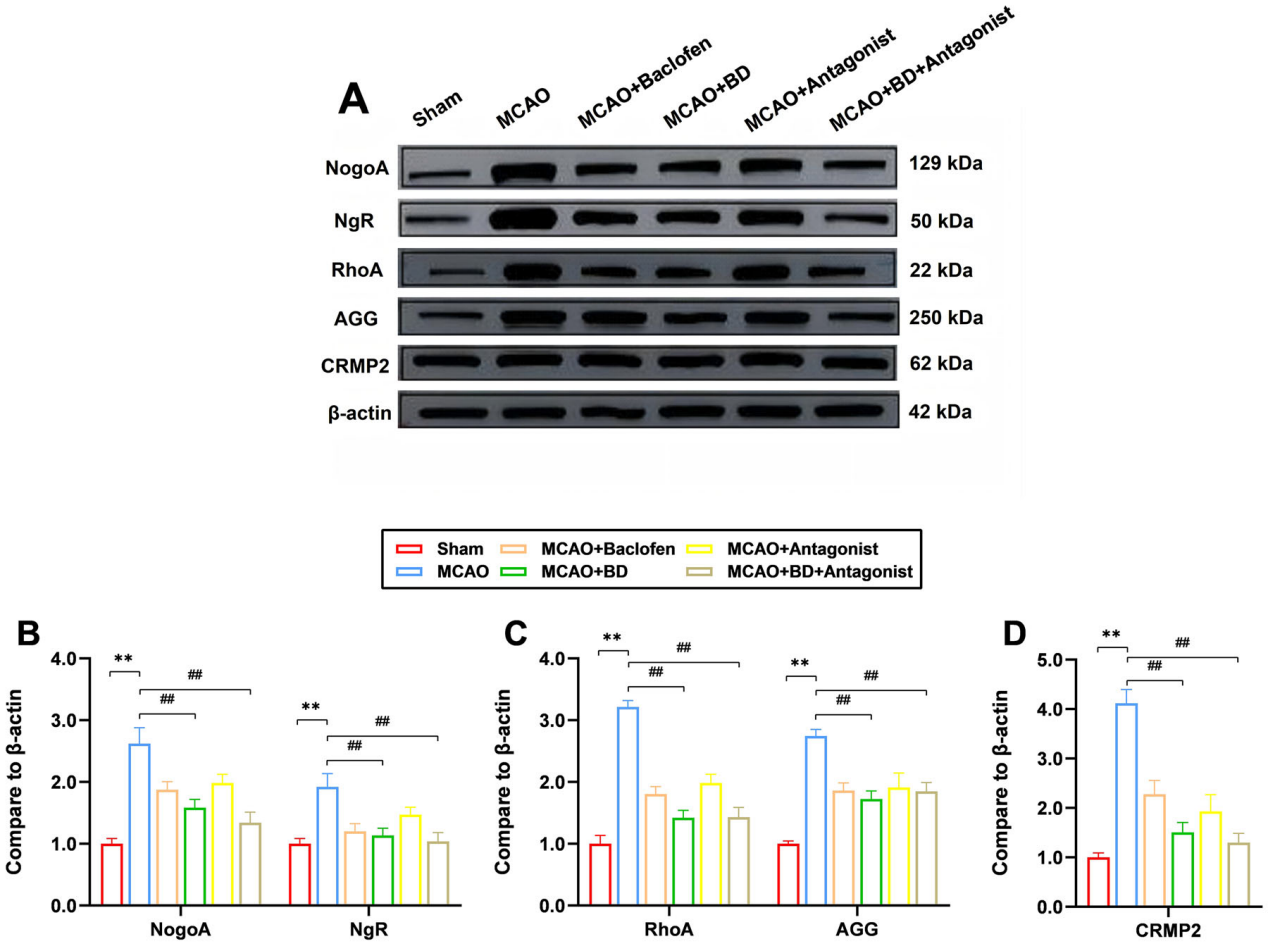

Olig2, a key marker of oligodendrocyte‐lineage cells (including oligodendrocyte precursor cells), regulates differentiation, myelination, and post‐injury repair. NgR is a well‐characterized mediator of inhibitory signaling in the central nervous system, traditionally associated with neuronal suppression of axonal regeneration and synaptic plasticity through activation of the RhoA/ROCK pathway. However, emerging evidence suggests that NgR may also play a functional role in glial cells. Therefore, we performed co‐immunofluorescence staining for NgR and Olig2 to comprehensively investigate the multifaceted mechanisms by which BD promotes synaptic remodeling and functional recovery in rats with PSS (Figure 8A). Our co‐localization analysis revealed a robust spatial overlap between NgR and Olig2 in the peri‐infarct regions of rats in the model group (Pearson's *R* > 0.5, Overlap *R* > 0.6, Mander's *R* > 0.5; Figure 8B), indicating that oligodendrocytes, not merely neurons, express NgR and potentially contribute to inhibitory microenvironment formation. Importantly, BD and BD+ antagonist treatments maintained similar co‐localization levels (Pearson's *R* > 0.5, Overlap *R* > 0.6, Mander's *R* > 0.5; Figure 8C). This dual effect suggested that BD not only targeted neuronal NgR/RhoA signaling, but also modulated oligodendrocyte‐specific NgR activity.

FIGURE 8Co‐immunofluorescence staining of NgR and Olig2. (**A**) Protein expression levels of NgR and Olig2 in the brain tissue around the infarct were detected using co‐immunofluorescence staining analysis, 4 weeks after the intervention; right, pixel intensity plot for co‐immunofluorescence staining. (**B**) Co‐localized expression levels of NgR and Olig2 in the brain tissue around the infarct were detected by co‐immunofluorescence staining 4 weeks after intervention. (**C**) Co‐localization scores for NgR and Olig2 in each group (Pearson's *R* > 0.5, Overlap *R* > 0.6, Mander's *R* > 0.5) (*n* = 3).

**Figure:**
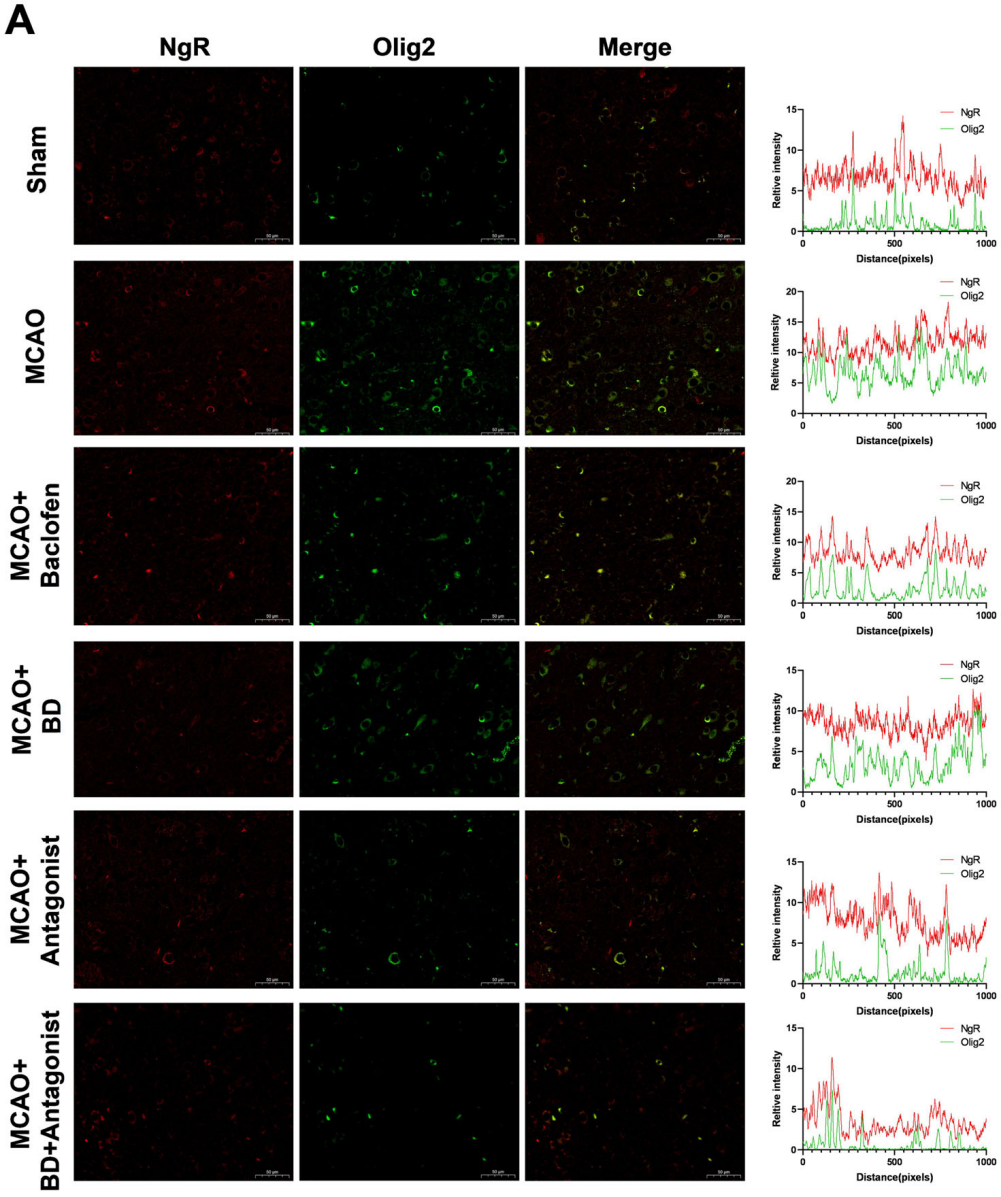

**Figure:**
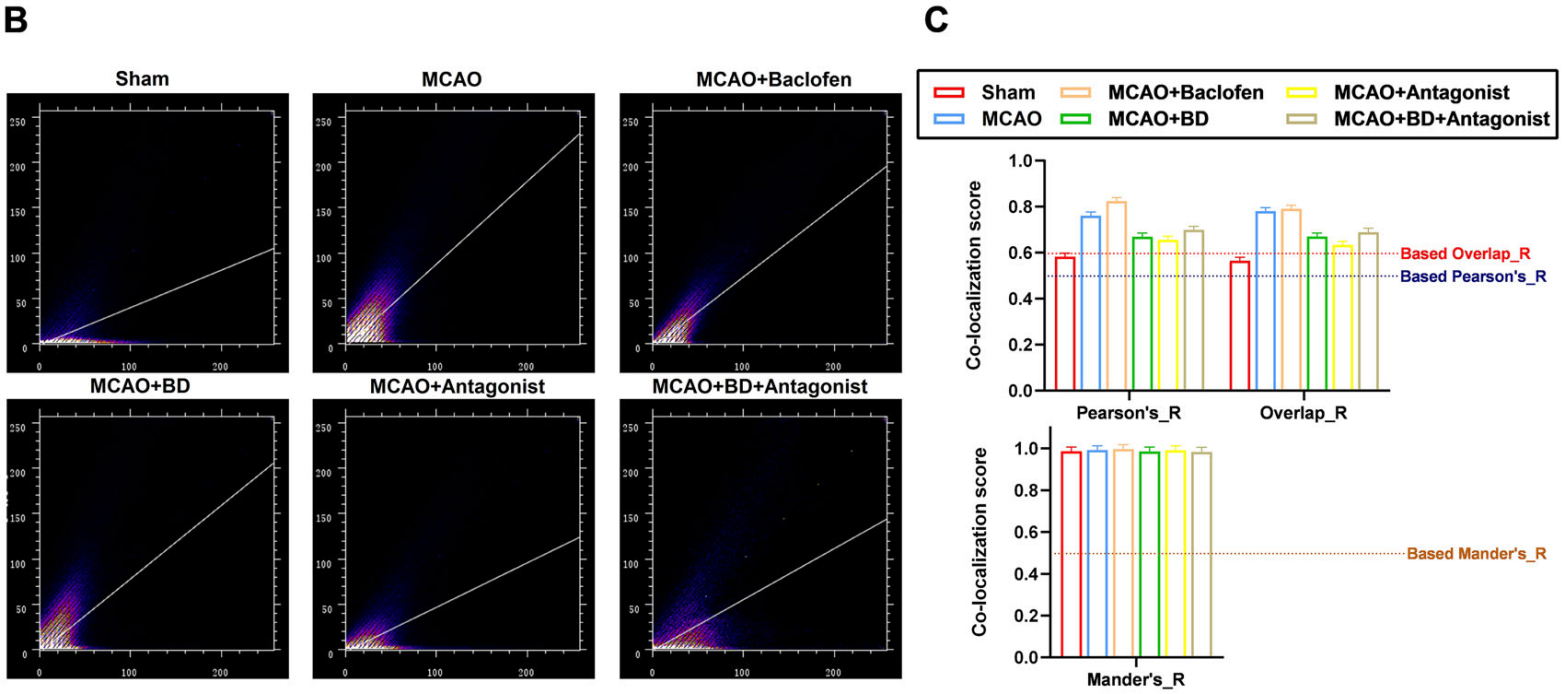

## 4 Discussion

PSS arises from ischemic damage to brain tissue, particularly the corticospinal tract (CST), originating from pyramidal neurons in the motor cortex. CST disruption weakens central inhibitory control over the spinal cord, leading to hyperactive stretch reflexes, increased muscle tone, and limb spasticity (Chen et al. 2025). While neurons lack regenerative capacity, synaptic connections can regenerate and restore neural control over spinal anterior horn cells to reduce stretch reflexes and alleviate spasticity (Yoon et al. 2021). This self‐repair mechanism primarily relies on synaptic plasticity and the dynamic structural and functional adaptation of synapses in response to activity, manifesting as changes in dendritic/axonal morphology and synaptic size and number (Lourenco et al. 2019; Lin et al. 2024). In this study, we demonstrated that BD improved motor and neurological functions in rats with PSS. BD enhanced synaptic plasticity by increasing PSD thickness and length, while narrowing synaptic clefts, as evidenced by morphological improvements in hemiplegic limbs. Notably, BD downregulated synaptic‐plasticity‐related proteins (Nogo‐A, NgR, RhoA, AGG, and CRMP2) in peri‐infarct brain tissue, suggesting that synaptic plasticity underpins the therapeutic efficacy of BD against PSS‐induced spasticity.

PSD, a disc‐shaped macromolecular assembly (width: hundreds of nanometers; thickness: ∼30–50 nm), comprises densely packed proteins that undergo dynamic assembly/disassembly in response to neuronal stimuli, directly influencing synaptic plasticity (Zeng et al. 2018). TEM revealed blurred synaptic ultrastructure, reduced PSD thickness, shortened PSD length, and widened synaptic clefts in MCAO‐model rats. Both BD and BD+ antagonist treatments restored PSD morphology (thickness and length) and narrowed synaptic clefts, indicating the protective role of BD in preserving synaptic plasticity at the ultrastructural level. These findings highlight the capacity of BD to counteract pathological synaptic remodeling, offering a morphological foundation for its therapeutic benefits in PSS.

Nogo‐A, currently recognized as the most potent myelin‐associated protein that inhibits axonal regeneration (Nagaraj et al. 2020), binds to its receptor, NgR, following cerebral infarction, thereby obstructing the structural and functional recovery of the CST, suppressing axonal regeneration, and restricting synaptic plasticity (Lin et al. 2019; Tang 2020). Anti‐Nogo‐A therapy enhances contralateral axonal sprouting across the midline, thereby promoting neural repair (Wahl et al. 2020). After neuronal injury, both the mRNA and protein levels of Nogo‐A are upregulated, and proteolytic cleavage generates bioactive fragments. These fragments are predominantly secreted via exosomes and bind to receptors to activate downstream signaling pathways, ultimately inducing growth cone collapse and impairing synaptic plasticity (Nheu et al. 2022). Our results demonstrated that BD and BD+ antagonist treatments significantly reduced Nogo‐A expression levels in peri‐infarct brain tissue compared to those in the model group, indicating the capacity of BD to attenuate Nogo‐A‐mediated inhibition and facilitate neural regeneration in rats with PSS.

Members of the NgR family are pivotal regulators of synaptic assembly and plasticity. NgR plays a critical role in determining the homeostatic set point for experience‐dependent synaptic scaling and modulating synaptic plasticity by suppressing dynamic synaptic alterations in the adult brain (Akbik et al. 2013). Notably, within the perforant pathway, a key circuit for synaptic plasticity and cognitive function, NgR knockdown reduces neuronal death and enhances synaptic efficacy (Jiang et al. 2023). NgR governs both the formation/stabilization of synaptic morphology and the regulation of axonal growth and elongation, rendering therapies targeting NgRs a major focus for promoting axonal regeneration (Xu et al. 2020). Our findings revealed that BD and BD+ antagonist treatment significantly reduced NgR expression levels in peri‐infarct brain tissue, suggesting that BD promotes neural growth and enhances synaptic plasticity. Notably, in the Western blot results, the relatively modest inhibitory effect of MCAO + NgR antagonist treatment on NgR levels may be attributed to the mechanism of NEP1‐40—a competitive peptide antagonist that binds to NgR to block Nogo‐A signaling while exerting minimal impact on receptor expression.

RhoA, a key regulator of the actin cytoskeleton, plays an essential role in coordinating synaptic development and remodeling (Hall 2012). Its function in cytoskeletal dynamics is largely mediated by the activation of Rho kinase (ROCK), which inhibits neurite outgrowth (Han et al. 2020). In neurons, the Rho GTPase Rac1 promotes axonal and dendritic growth, as well as the formation and maintenance of dendritic spines and synapses, whereas RhoA induces axonal/dendritic retraction and spine/synapse loss (Ng and Luo 2004). RhoA is also critically involved in central nervous system (CNS) injuries. It is strongly upregulated and activated following brain or spinal cord trauma, leading to growth cone collapse and axonal regeneration failure (Hu et al. 2017; Seo et al. 2022). Mechanistically, Rho‐guanine nucleotide exchange factors (Rho‐GEFs) couple with Nogo‐A to form the Rho GEF‐LARG complex, which activates the GTPase RhoA and mediates Rho‐ROCK‐dependent cellular effects that suppress the regeneration of damaged axons (Tang 2020). Our results demonstrated that BD and BD+ antagonist treatments significantly reduced RhoA expression levels in peri‐infarct brain tissue compared with those in the model group, indicating the potential of BD to attenuate RhoA activity and promote neural regeneration in rats with PSS.

AGG, a core member of the chondroitin sulfate proteoglycan (CSPG) family, contributes to the structural stability of the ECM and synaptic plasticity through its chondroitin sulfate chains. CSPGs are critical ECM components that serve as guidance cues during neural development and maintain CNS homeostasis in the intact brain (Gupta et al. 2025). However, CNS injury upregulates CSPG production across multiple cell types, leading to the formation of CSPG‐rich glial scars and ECM deposition in the peri‐lesional regions (Pu et al. 2018; Tran et al. 2022). While CSPGs in glial scars act as physical barriers to confine injury spread (Djerbal et al. 2017; Sami et al. 2020), they simultaneously inhibit axonal regeneration by binding to protein tyrosine phosphatase sigma (PTPσ), disrupting autophagosome‐lysosome fusion, and destabilizing axonal growth cone homeostasis (Kurihara et al. 2017). Our findings demonstrated that BD and BD+ antagonist treatments significantly reduced AGG expression levels in peri‐infarct brain tissue compared to those in the model group, suggesting that BD attenuates AGG levels to enhance synaptic plasticity and promote neural regeneration in rats with PSS.

CRMP2, a foundational member of the CRMP family, functions as a critical mediator protein involved in axonal guidance and the response to inhibitory cues, such as collapsins (Nakamura et al. 2020). The aberrant phosphorylation of CRMP2 induces cytoskeletal instability, impairs axonal transport, and increases neuronal susceptibility to stress (Wang and Ohshima 2024). Phosphorylated CRMP2, along with myelin‐associated inhibitors and semaphorins, accumulates at scar tissue sites to suppress axonal growth and regeneration (Filbin 2003). Mechanistically, Nogo‐66 binding to NgR1 recruits PlexinA2 as a coreceptor, facilitating the assembly of a ternary NgR1/PlexinA2/CRMP2 complex. This complex potentiates Rho‐GTP synthesis, activating downstream ROCK to dephosphorylate cofilin, thereby inhibiting actin filament polymerization and blocking the regeneration of injured axons (Sekine et al. 2019). Our current findings demonstrated that BD enhances synaptic plasticity and promotes neurological recovery in rats with PSS by reducing AGG levels.

Although NgR has traditionally been considered neuron‐restricted, studies confirm its functional expression in oligodendrocyte lineage cells under pathological conditions. Oligodendrocyte precursor cells (OPCs) exhibit endogenous NgR expression, and its overexpression impedes differentiation and remyelination (Tang et al. 2007). In rat CNS injury models, oligodendrocytes upregulate NgR, forming an autocrine OMgP/NgR signaling cascade that potentiates inhibitory ECM deposition and impedes repair (Guo et al. 2007). Our study revealed that the co‐localization of NgR and Olig2 reveals that oligodendrocytes are potential key mediators of the therapeutic effects of BD. By inhibiting NgR signaling in these cells, BD alleviates inhibitory ECM remodeling and promotes synaptic plasticity, with antagonist combination therapy conferring additional benefits. This mechanism synergizes with the direct effects of BD on neuronal proteins, collectively enhancing its therapeutic efficacy. Notably, while the BD+ antagonist treatment reduced NgR/Olig2 co‐localization, it increased AGG expression levels compared with those observed after BD monotherapy (Figure 7). This finding may reflect compensatory ECM stabilization by AGG to counteract excessive synaptic destabilization, underscoring the delicate balance between plasticity and stability during recovery.

In conclusion, this study demonstrated that BD ameliorated modified mNSS, limb spasticity, and motor function in rats with PSS by increasing PSD thickness and length; narrowing synaptic clefts; and downregulating the synaptic‐plasticity‐related proteins Nogo‐A, NgR, RhoA, AGG, and CRMP2 in peri‐infarct brain tissue. Notably, BD suppressed oligodendrocyte‐derived NgR signaling to alleviate ECM‐mediated inhibition, while promoting synaptic remodeling. The synergistic efficacy of the BD+ antagonist treatment further underscores the therapeutic potential of dual pathway modulation. These findings suggest that BD is a multi‐target intervention that bridges neuronal and glial interactions during recovery from PSS. However, some limitations remain. First, causal relationships with NgR/Olig2 co‐localization require validation through conditional‐knockout models (e.g., Olig2‐Cre/NgR‐flox) to confirm cell‐specific roles. Second, hormonal influences were not addressed because of the exclusive use of male rats, despite known sex‐dependent differences in stroke pathophysiology (Levy et al. 2023). Finally, longitudinal tracking of synaptic protein dynamics is needed to resolve stage‐specific mechanisms underlying recovery.

## Author Contributions

**Xiongxing Sun**: Writing – original draft, Writing – review & editing, Data curation, Formal analysis, Resources, Software, Visualization, Funding acquisition. **Shanshan Zeng**: Writing – review & editing, Data curation, Methodology, Project administration. **Shigao Lin**: Writing – original draft, Software, Visualization, Formal analysis. **Lingying Wu**: Writing – original draft, Data curation, Visualization. **Xukun Tang**: Writing – original draft, Conceptualization, Formal analysis. **Jiajian Zhu**: Writing – original draft, Formal analysis, Software. **Yuhui Zhang**: Writing – original draft, Data curation. **Lu Li**: Writing – original draft, Investigation. **Ziming Chen**: Writing – original draft, Validation, Software. **Xinyu Deng**: Writing – original draft, Visualization, Resources. **Dahua Wu**: Supervision, Writing – review & editing, Funding acquisition. **Le Xie**: Writing – original draft, Writing – review & editing, Funding acquisition, Supervision.

## Conflicts of Interest

The authors declare that the research was conducted in the absence of any commercial or financial relationships that could be construed as a potential conflict of interest.

## Funding

This study was supported by National Natural Science Foundation of China General Program (No. 8237153821); Hunan Province Science and Technology Innovation Plan Project (No. 2023RC3215); Hunan Province Health Commission Research Program Project (No. B202303077689); Hunan Innovative Province Construction Special Project (No. 2023JJ40397); and Graduate Innovation Project of Hunan University of Chinese Medicine (No. 2024CX133).

## Supporting information



Supplementary Information

Supplementary Information

Supplementary Information

Supplementary Information

Supplementary Information

Supplementary Information

Supplementary Information

Supplementary Information

Supplementary Information

Supplementary Information

Supplementary Information

Supplementary Information

Supplementary Information

Supplementary Information

Supplementary Information

Supplementary Information

Supplementary Information

Supplementary Information

Supplementary Information

Supplementary Information

Supplementary Information

Supplementary Information

Supplementary Information

Supplementary Information

Supplementary Information

Supplementary Information

Supplementary Information

Supplementary Information

Supplementary Information

Supplementary Information

Supplementary Information

Supplementary Information

Supplementary Information

Supplementary Information

Supplementary Information

Supplementary Information

Supplementary Information

Supplementary Information

## Data Availability

The complete set of original Western blot images (three independent experimental replicates) supporting this study has been deposited as Supplementary Files 1–38. The data presented in the figures are derived from a representative experiment. Further information is available from the corresponding author upon reasonable request.
