# Supplementary figures and images for: Baishaoluoshi Decoction Mitigates Post‐Stroke Spasticity by Targeting Synaptic Plasticity Through the Nogo‐A/NgR Signaling Pathway
Source: Brain Behav. 2025 Dec 31;16(1):e71170. doi: 10.1002/brb3.71170 (PMC12755059; doi:10.1002/brb3.71170)

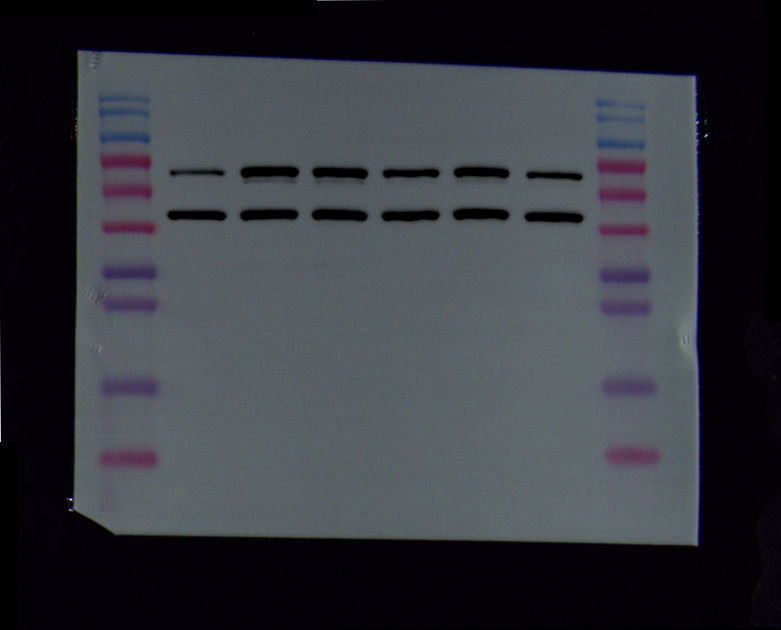

Supplement: Supplementary file 1 — Supplementary Information [file BRB3-16-e71170-s008.tif]

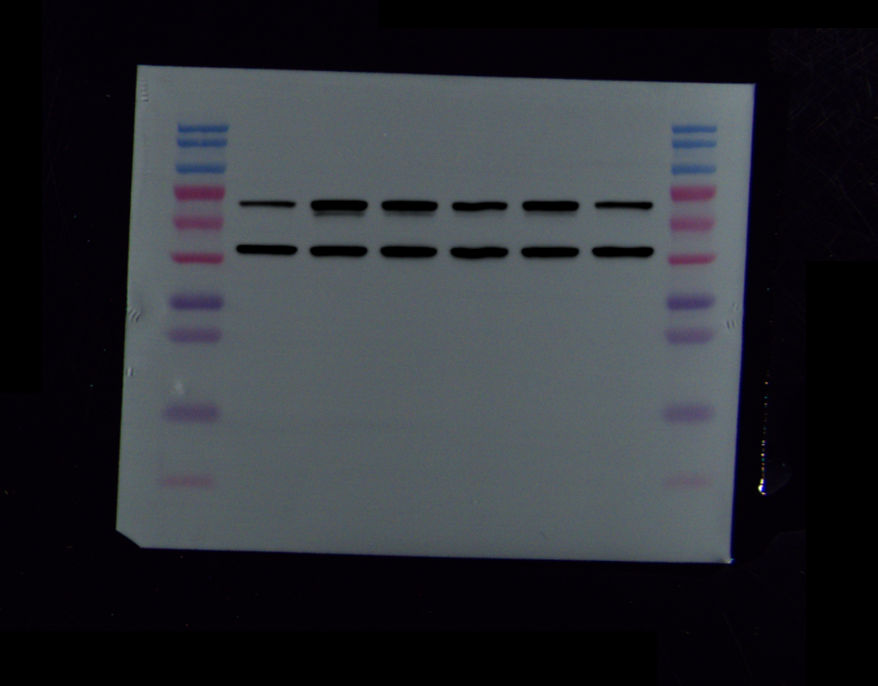

Supplement: Supplementary file 2 — Supplementary Information [file BRB3-16-e71170-s013.tif]

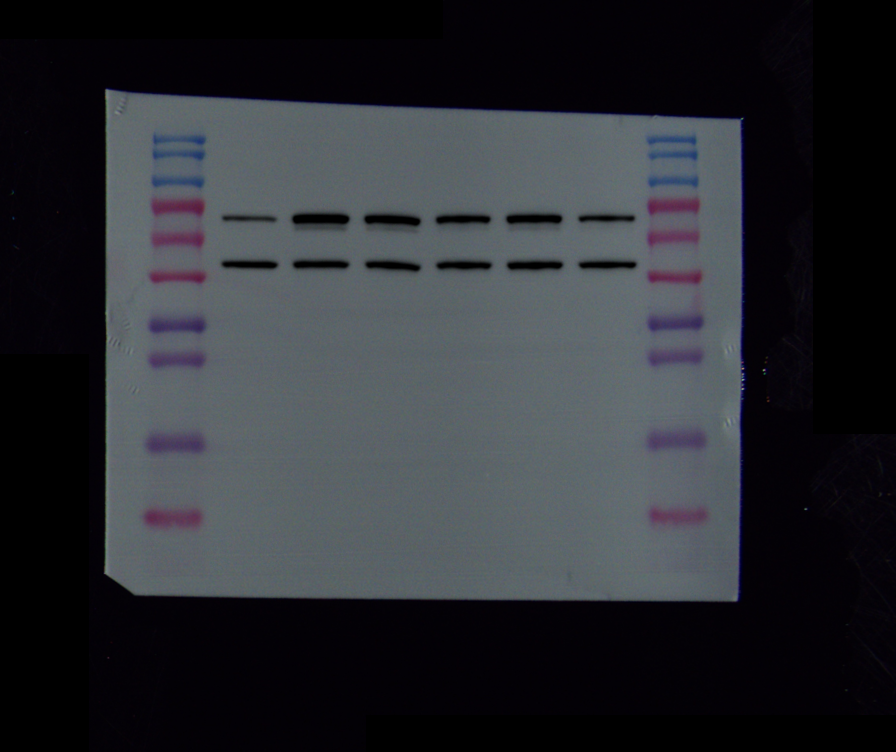

Supplement: Supplementary file 3 — Supplementary Information [file BRB3-16-e71170-s027.tif]

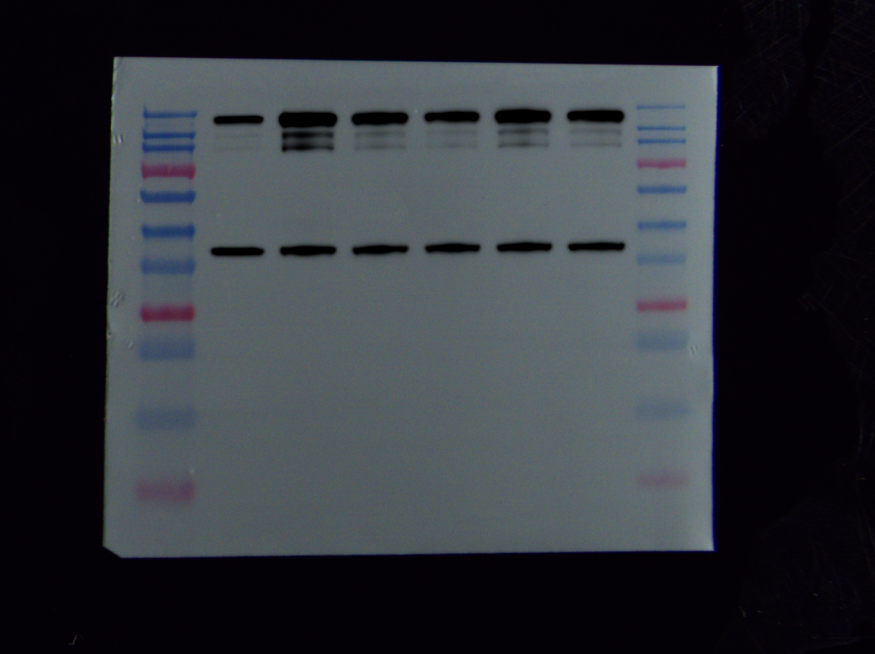

Supplement: Supplementary file 4 — Supplementary Information [file BRB3-16-e71170-s037.tif]

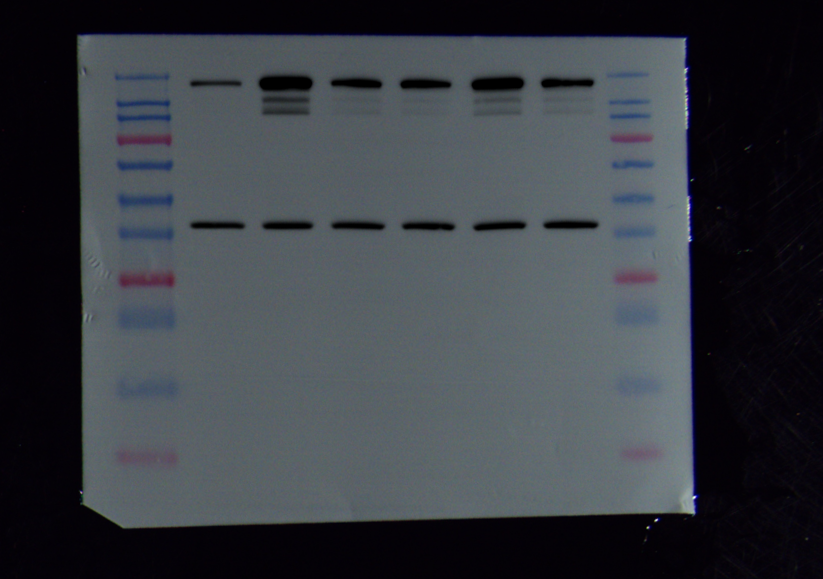

Supplement: Supplementary file 5 — Supplementary Information [file BRB3-16-e71170-s020.tif]

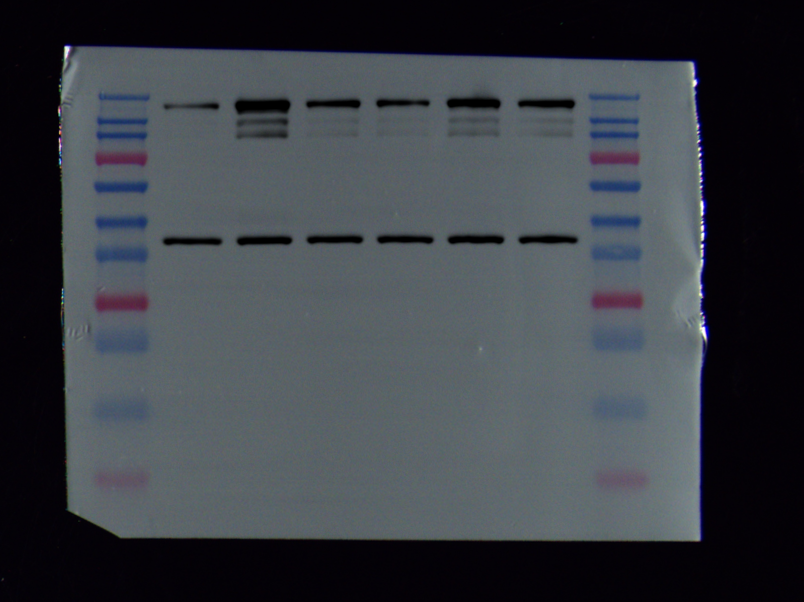

Supplement: Supplementary file 6 — Supplementary Information [file BRB3-16-e71170-s018.tif]

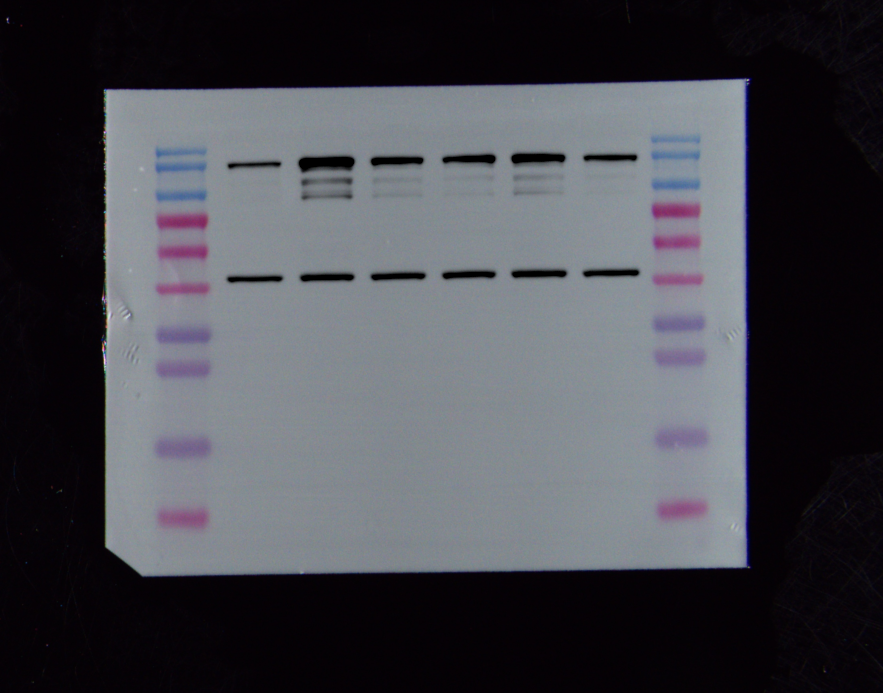

Supplement: Supplementary file 7 — Supplementary Information [file BRB3-16-e71170-s003.tif]

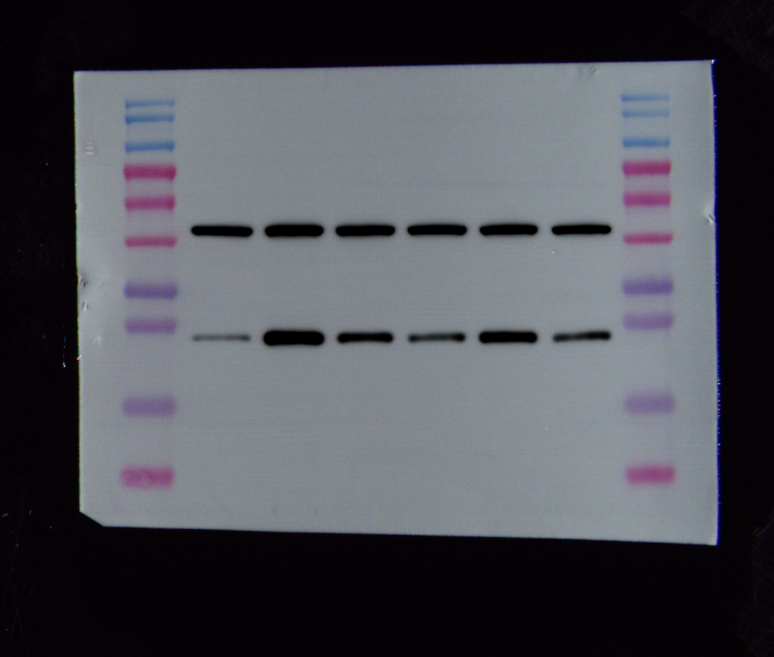

Supplement: Supplementary file 8 — Supplementary Information [file BRB3-16-e71170-s033.tif]

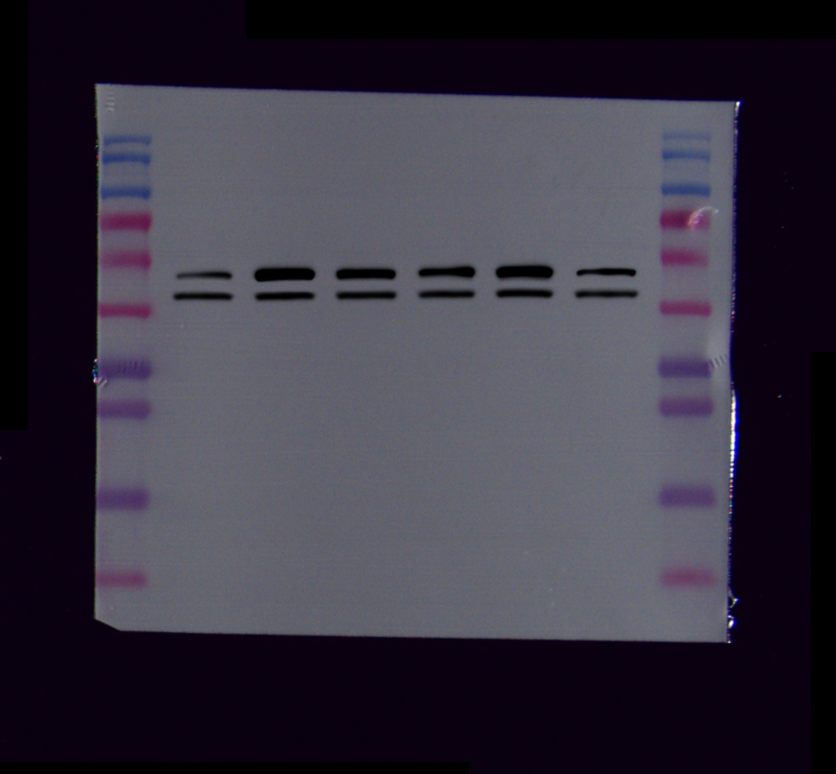

Supplement: Supplementary file 9 — Supplementary Information [file BRB3-16-e71170-s006.tif]

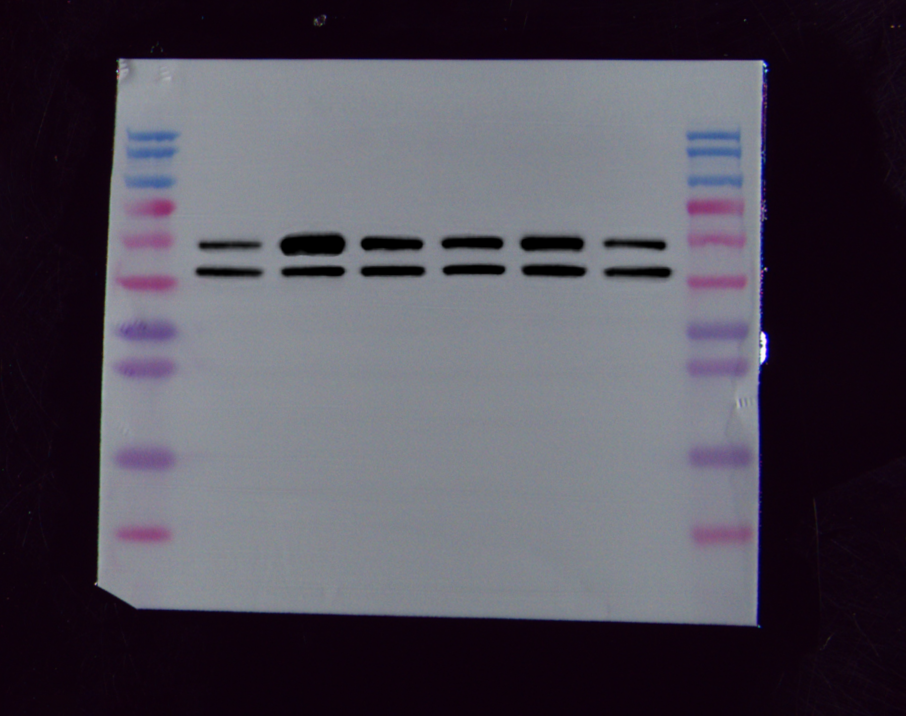

Supplement: Supplementary file 10 — Supplementary Information [file BRB3-16-e71170-s007.tif]

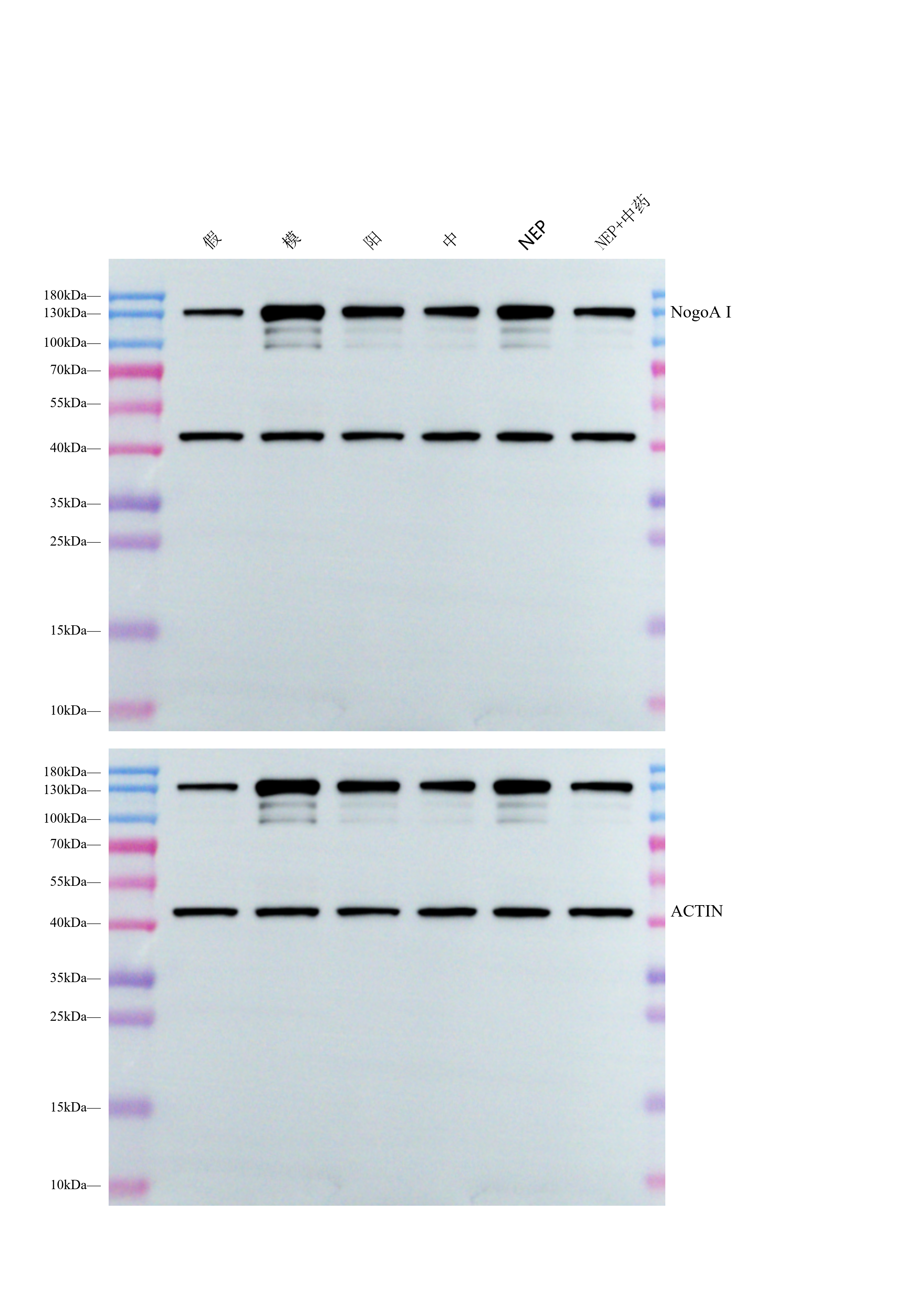

Supplement: Supplementary file 11 — Supplementary Information [file BRB3-16-e71170-s026.tif]

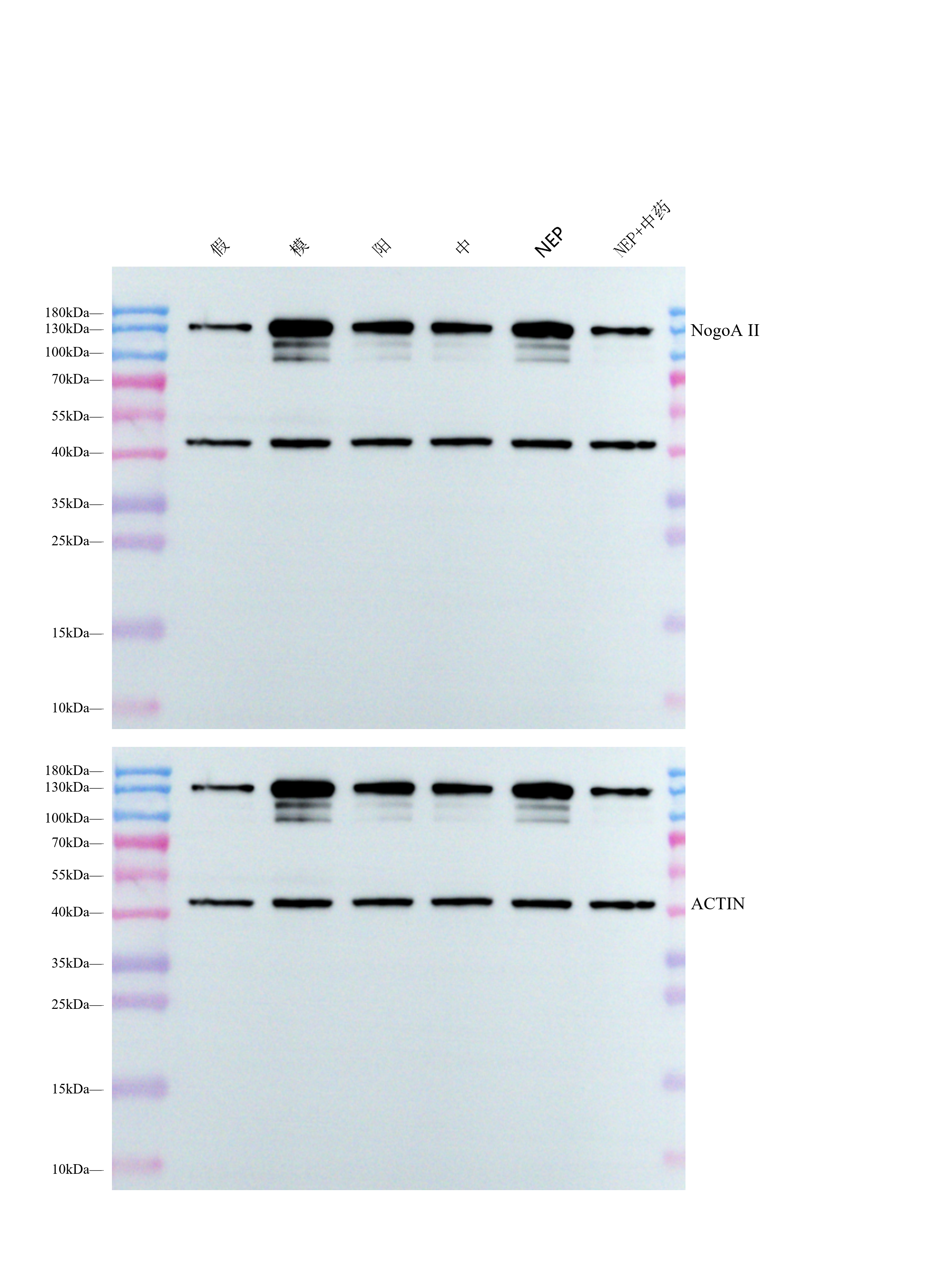

Supplement: Supplementary file 12 — Supplementary Information [file BRB3-16-e71170-s016.tif]

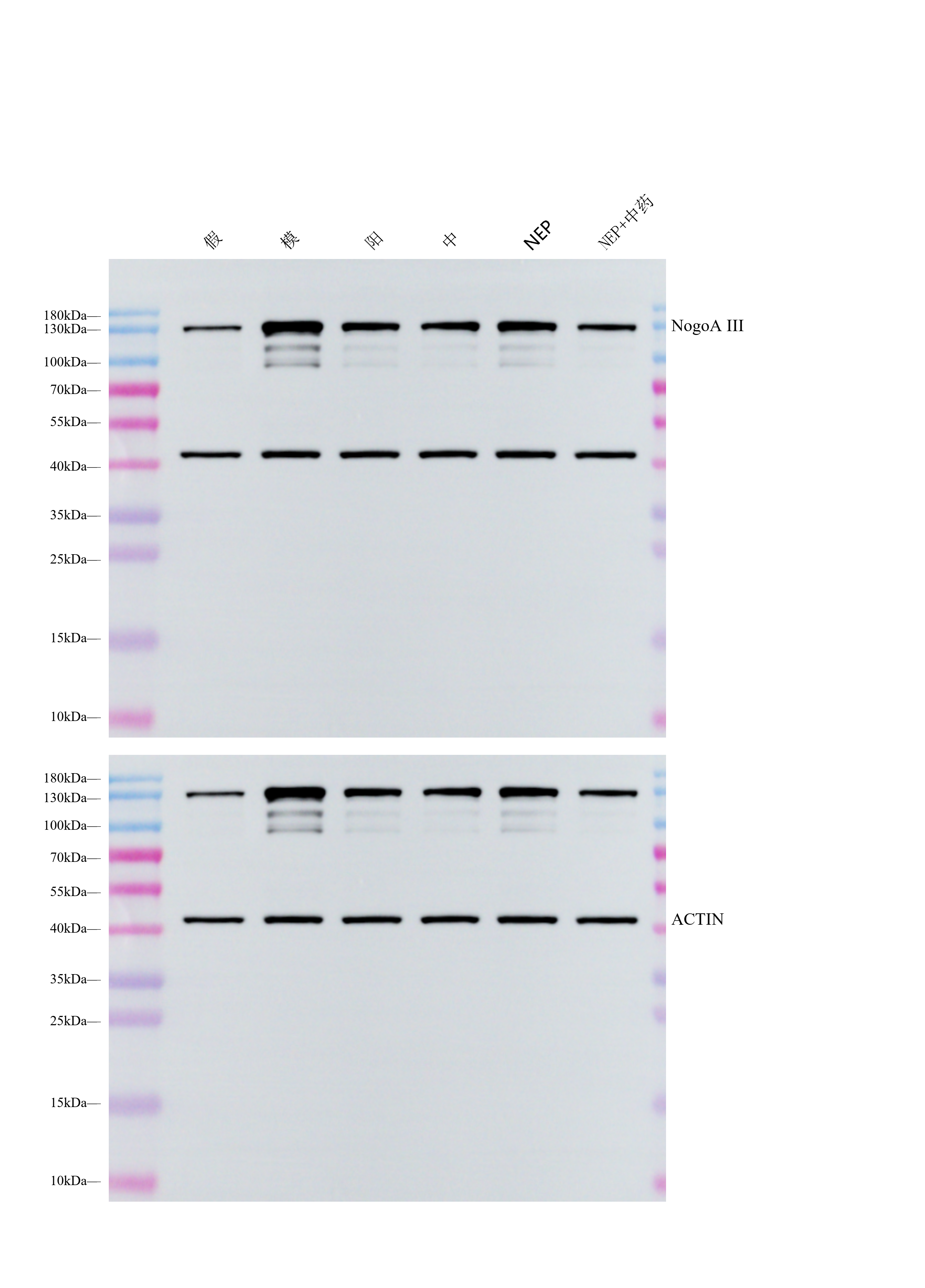

Supplement: Supplementary file 13 — Supplementary Information [file BRB3-16-e71170-s038.tif]

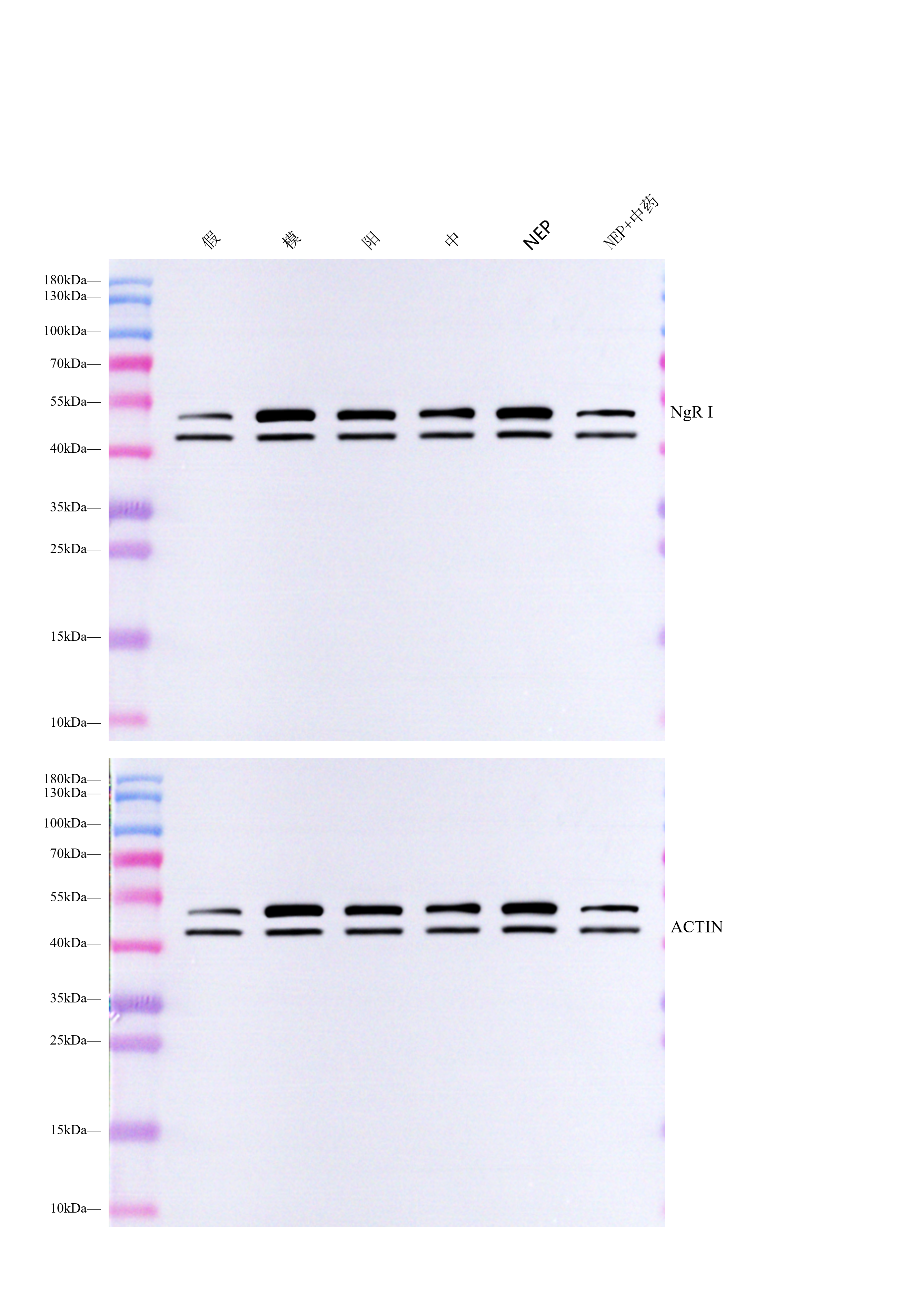

Supplement: Supplementary file 14 — Supplementary Information [file BRB3-16-e71170-s001.tif]

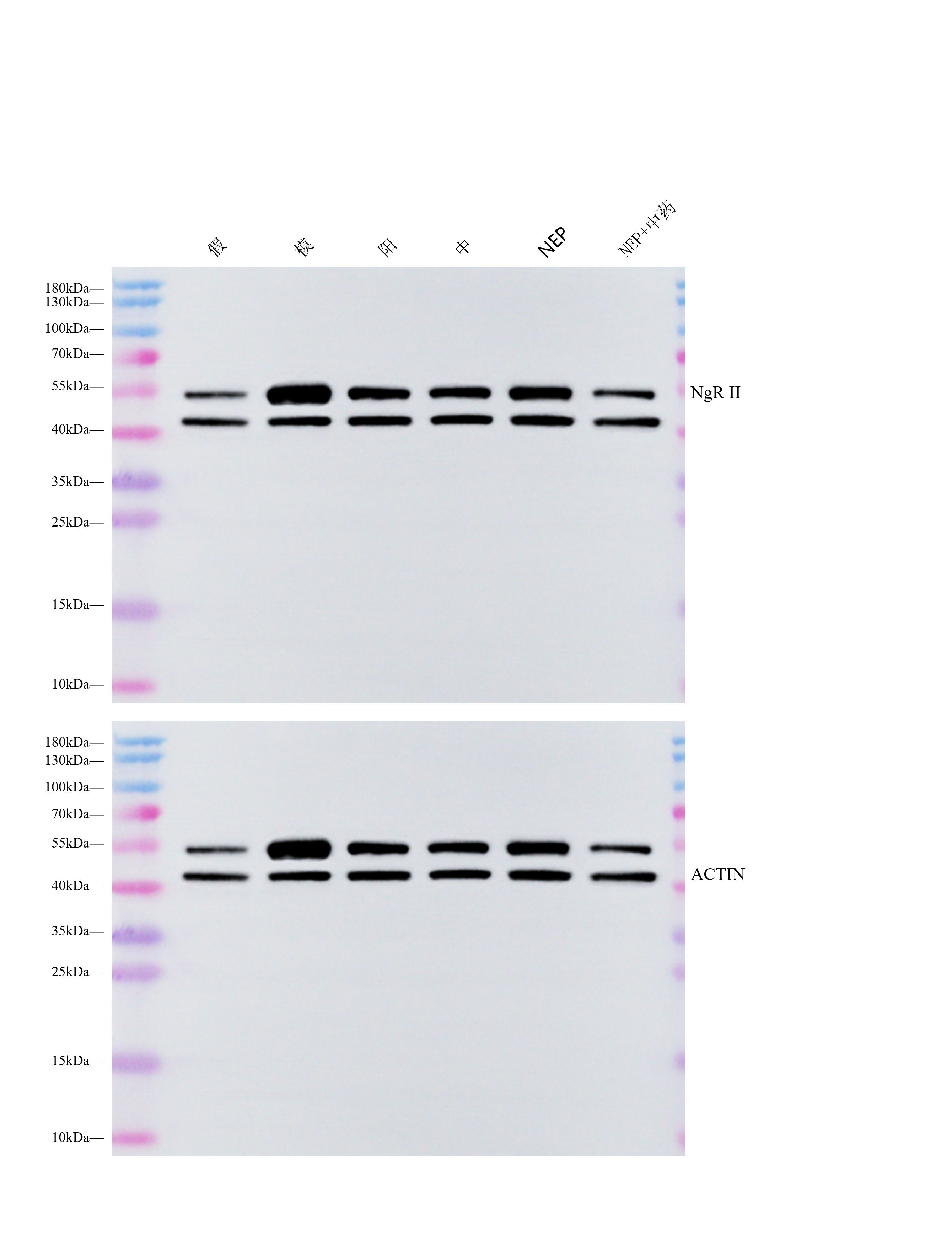

Supplement: Supplementary file 15 — Supplementary Information [file BRB3-16-e71170-s022.tif]

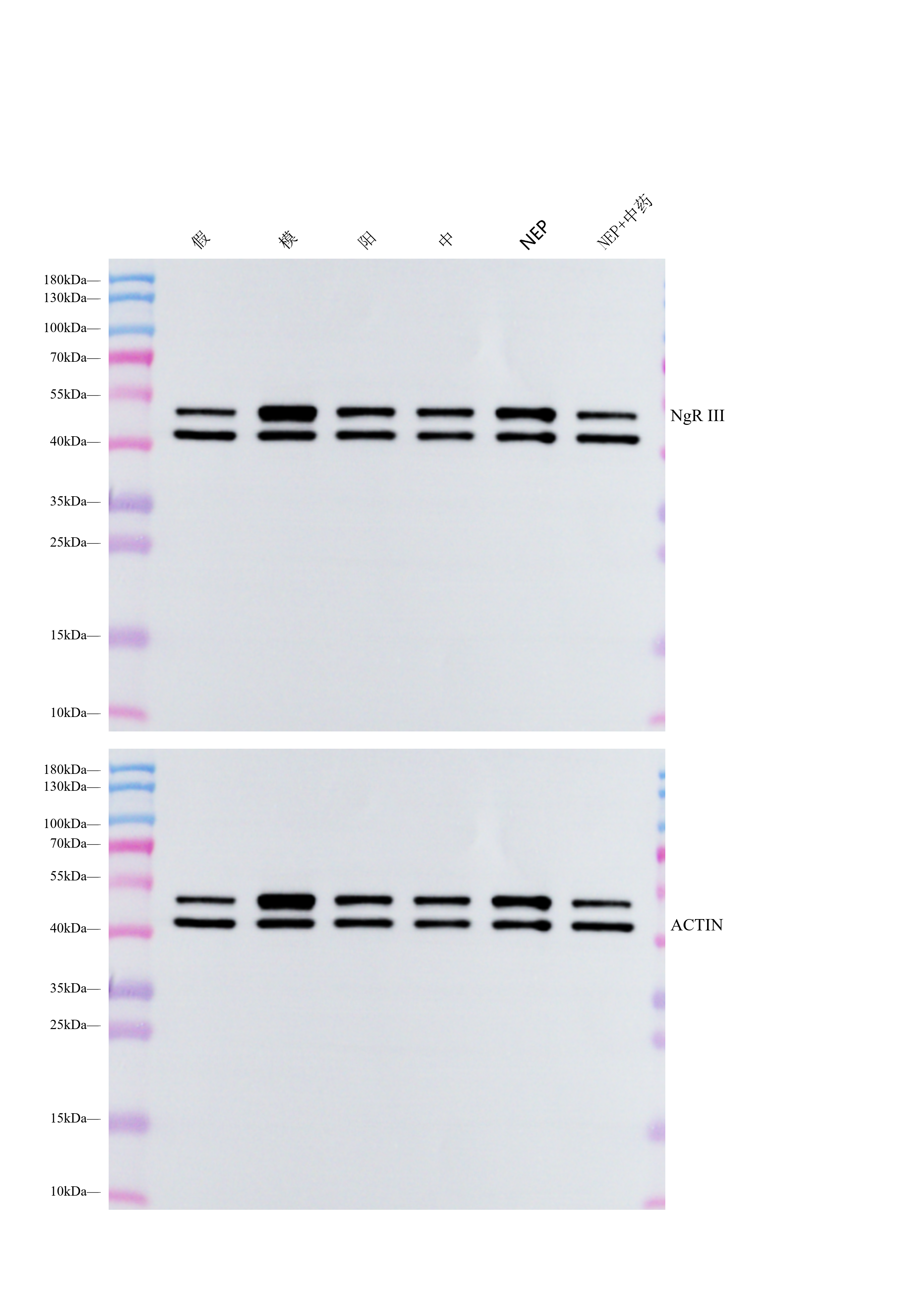

Supplement: Supplementary file 16 — Supplementary Information [file BRB3-16-e71170-s032.tif]

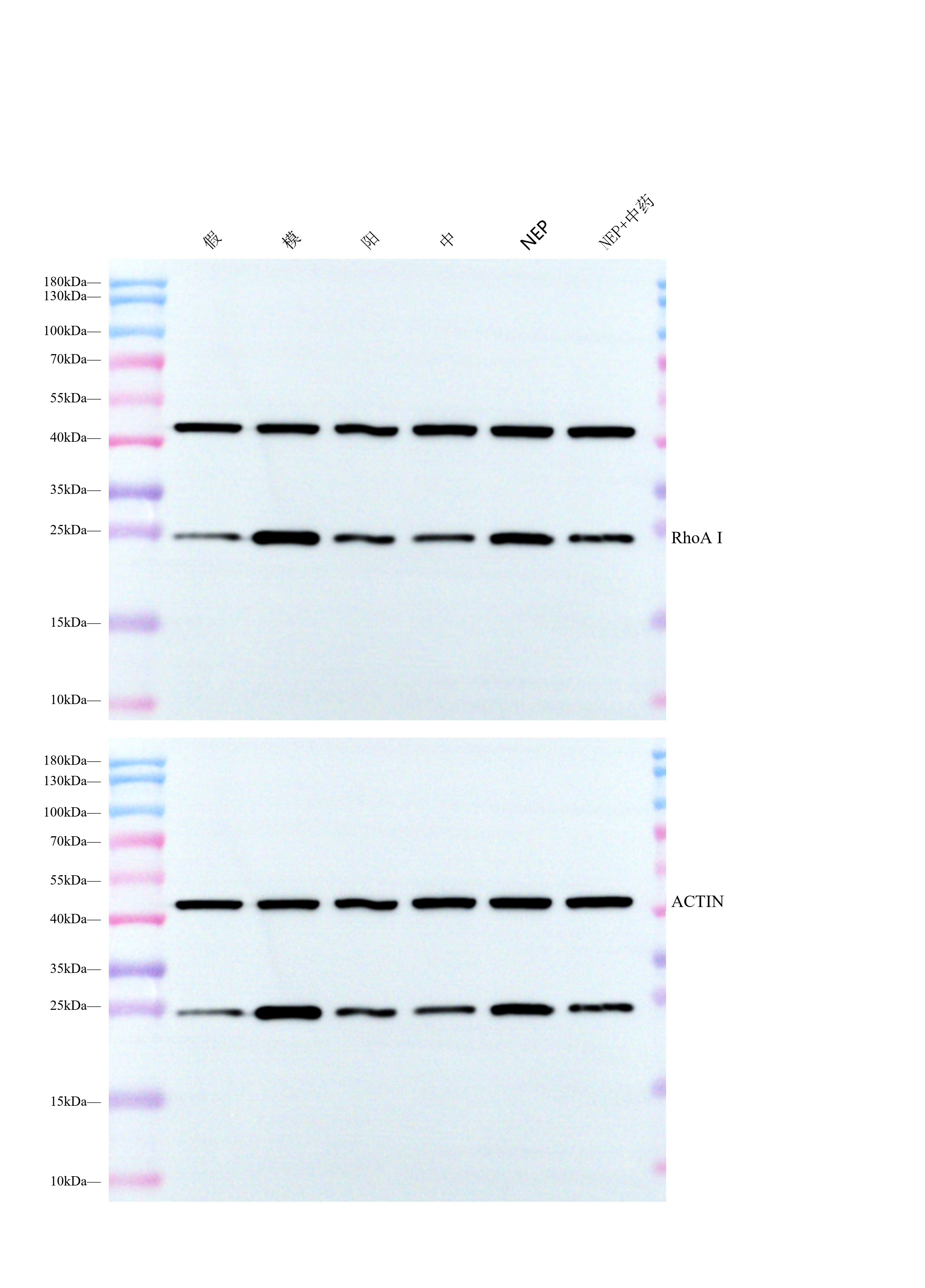

Supplement: Supplementary file 17 — Supplementary Information [file BRB3-16-e71170-s010.tif]

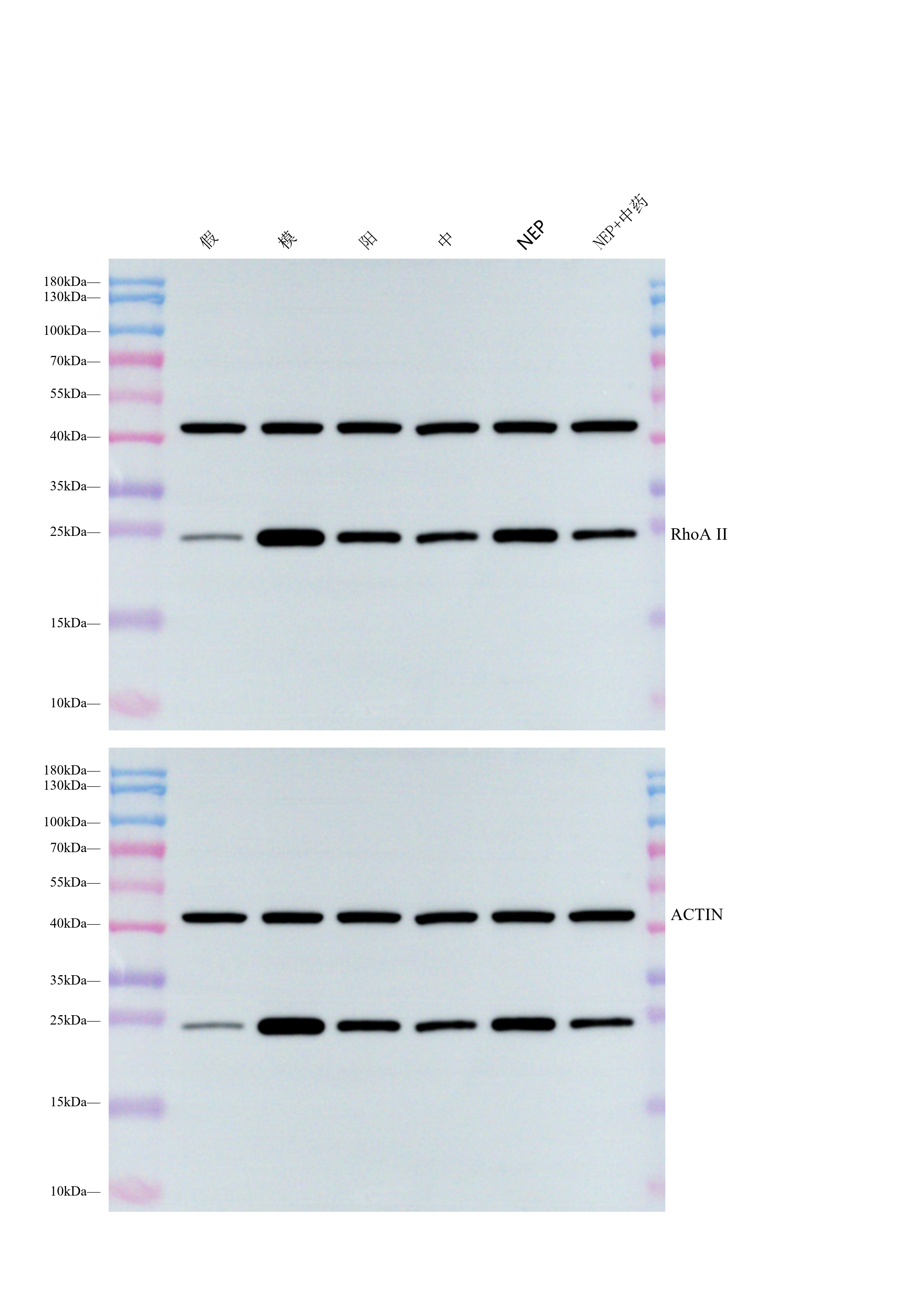

Supplement: Supplementary file 18 — Supplementary Information [file BRB3-16-e71170-s024.tif]

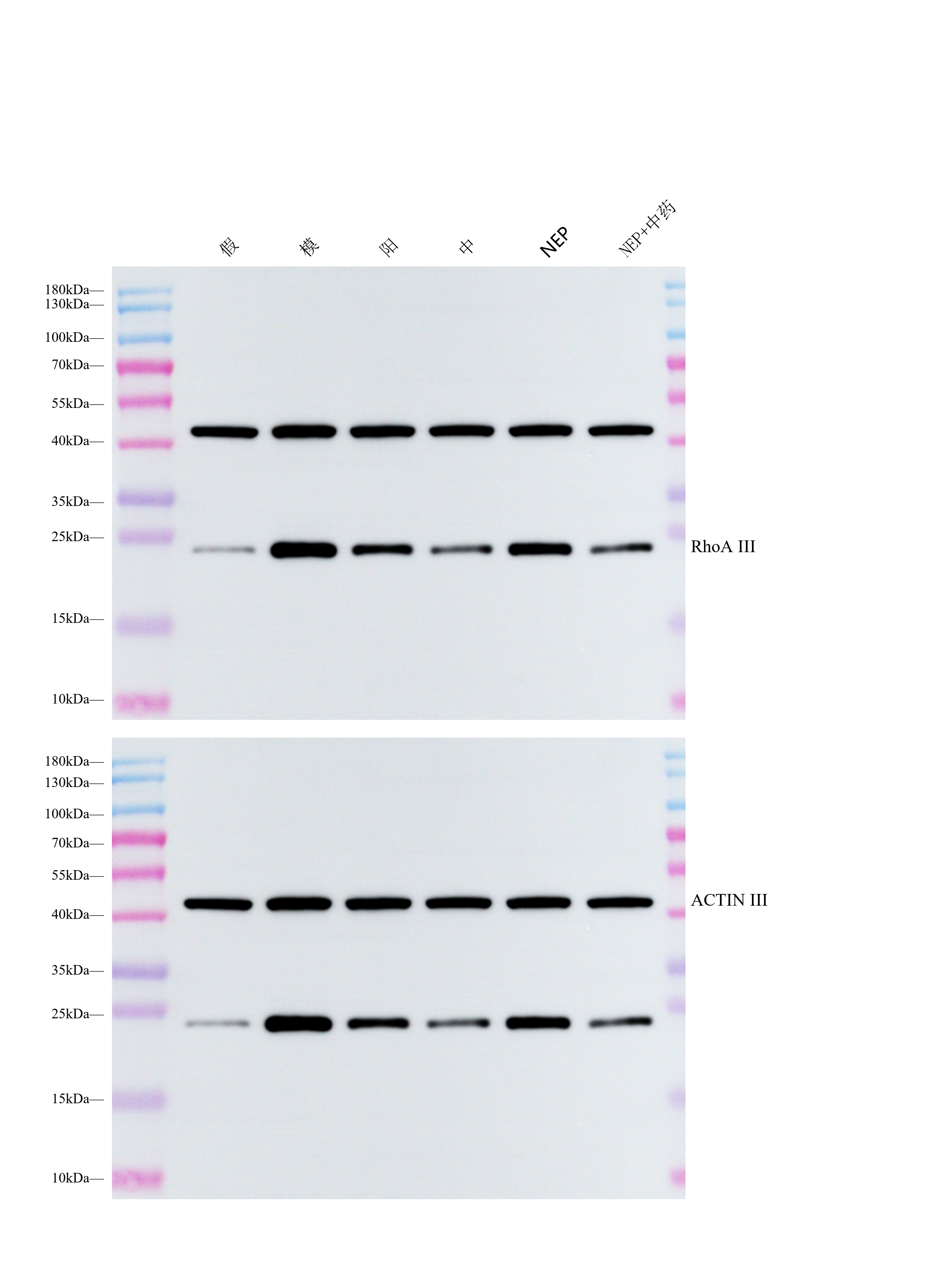

Supplement: Supplementary file 19 — Supplementary Information [file BRB3-16-e71170-s036.tif]

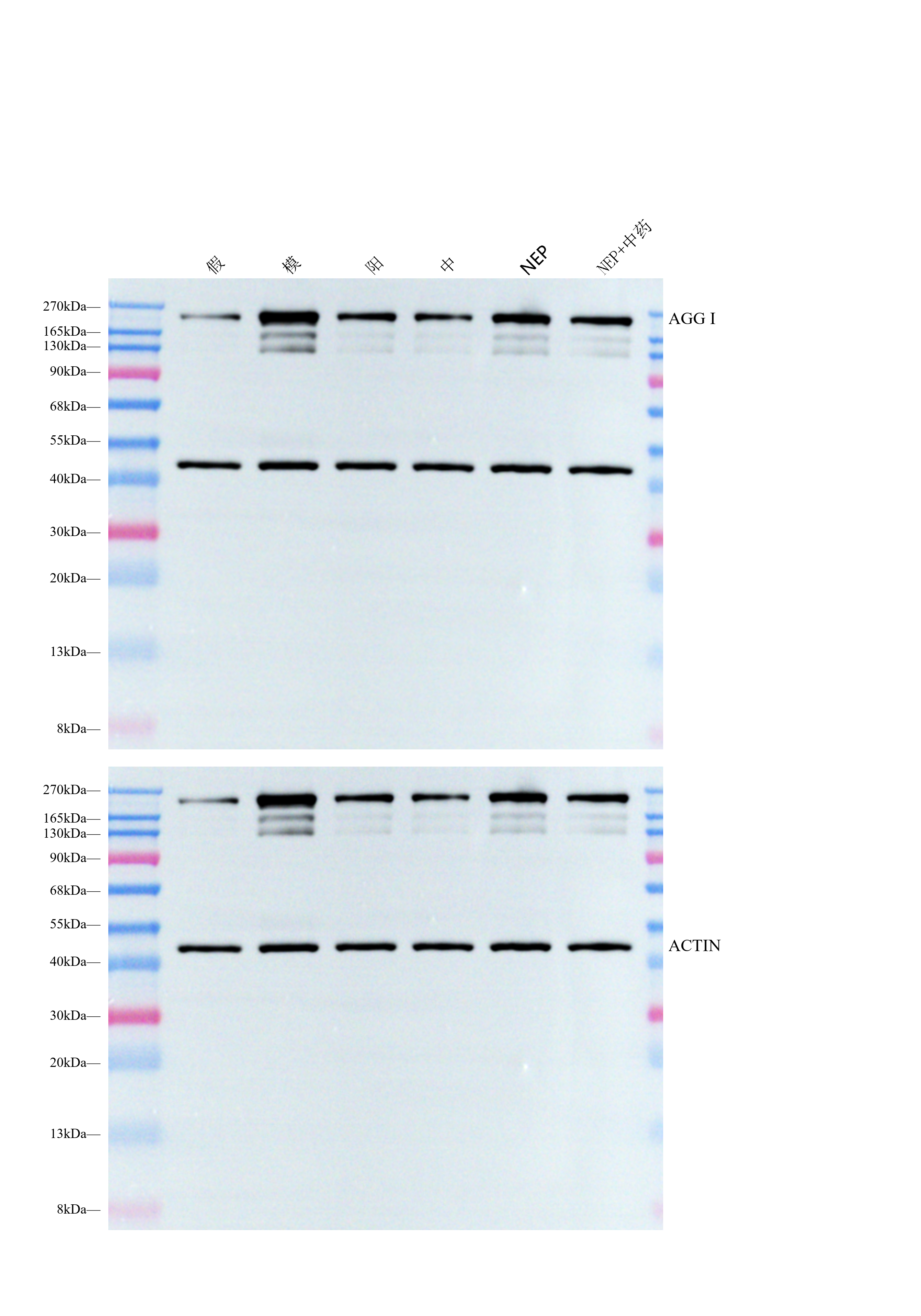

Supplement: Supplementary file 20 — Supplementary Information [file BRB3-16-e71170-s031.tif]

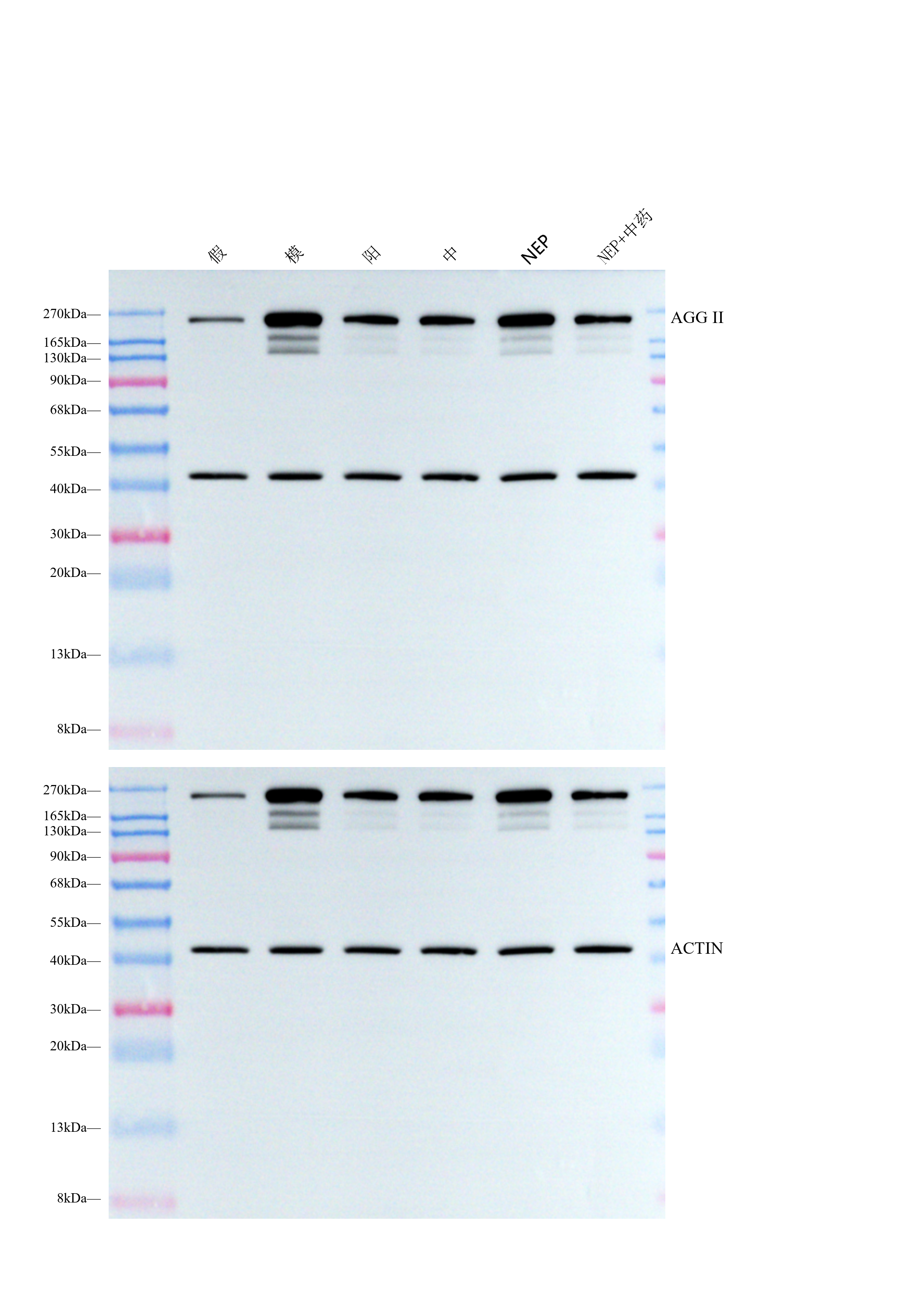

Supplement: Supplementary file 21 — Supplementary Information [file BRB3-16-e71170-s009.tif]

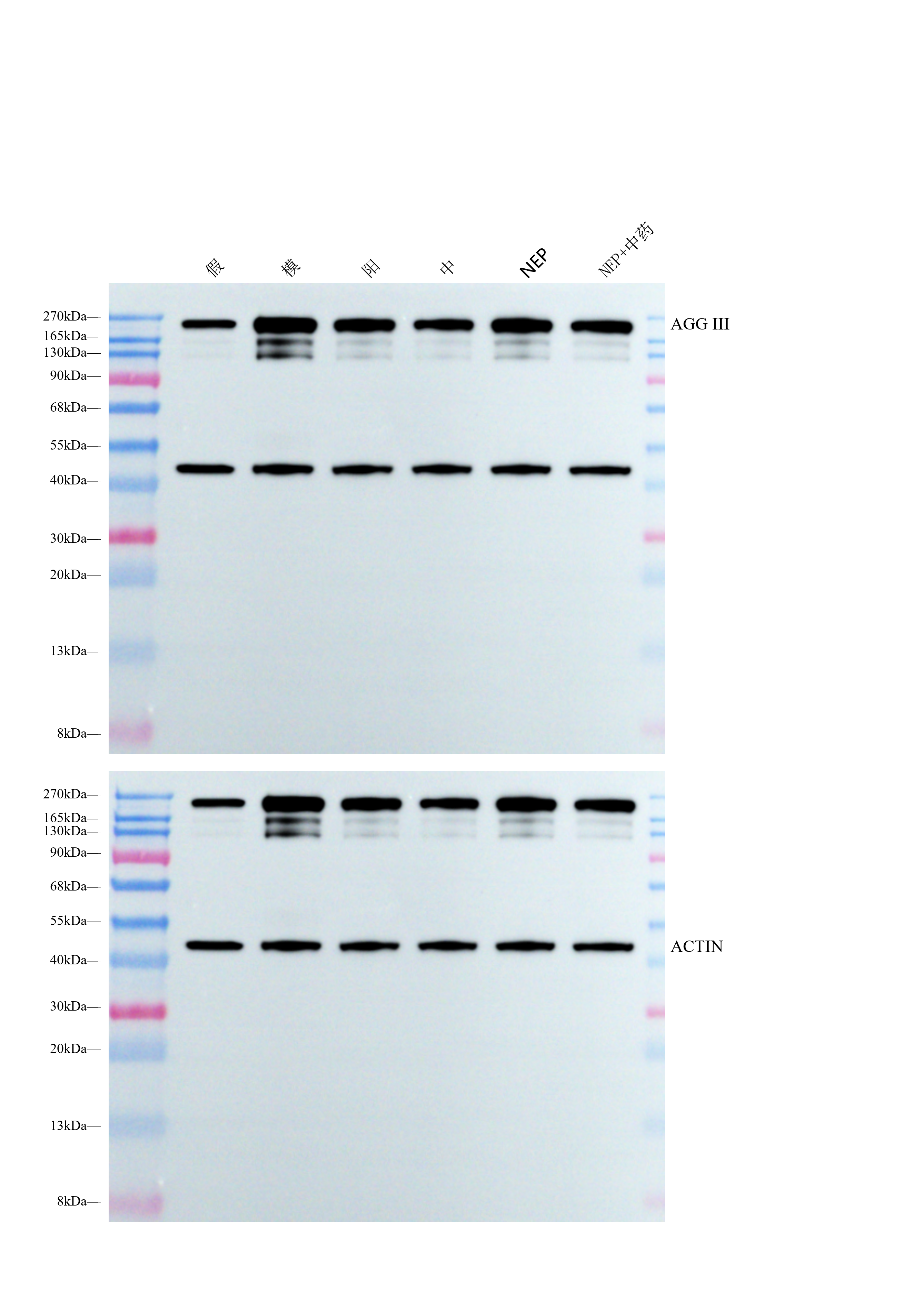

Supplement: Supplementary file 22 — Supplementary Information [file BRB3-16-e71170-s002.tif]

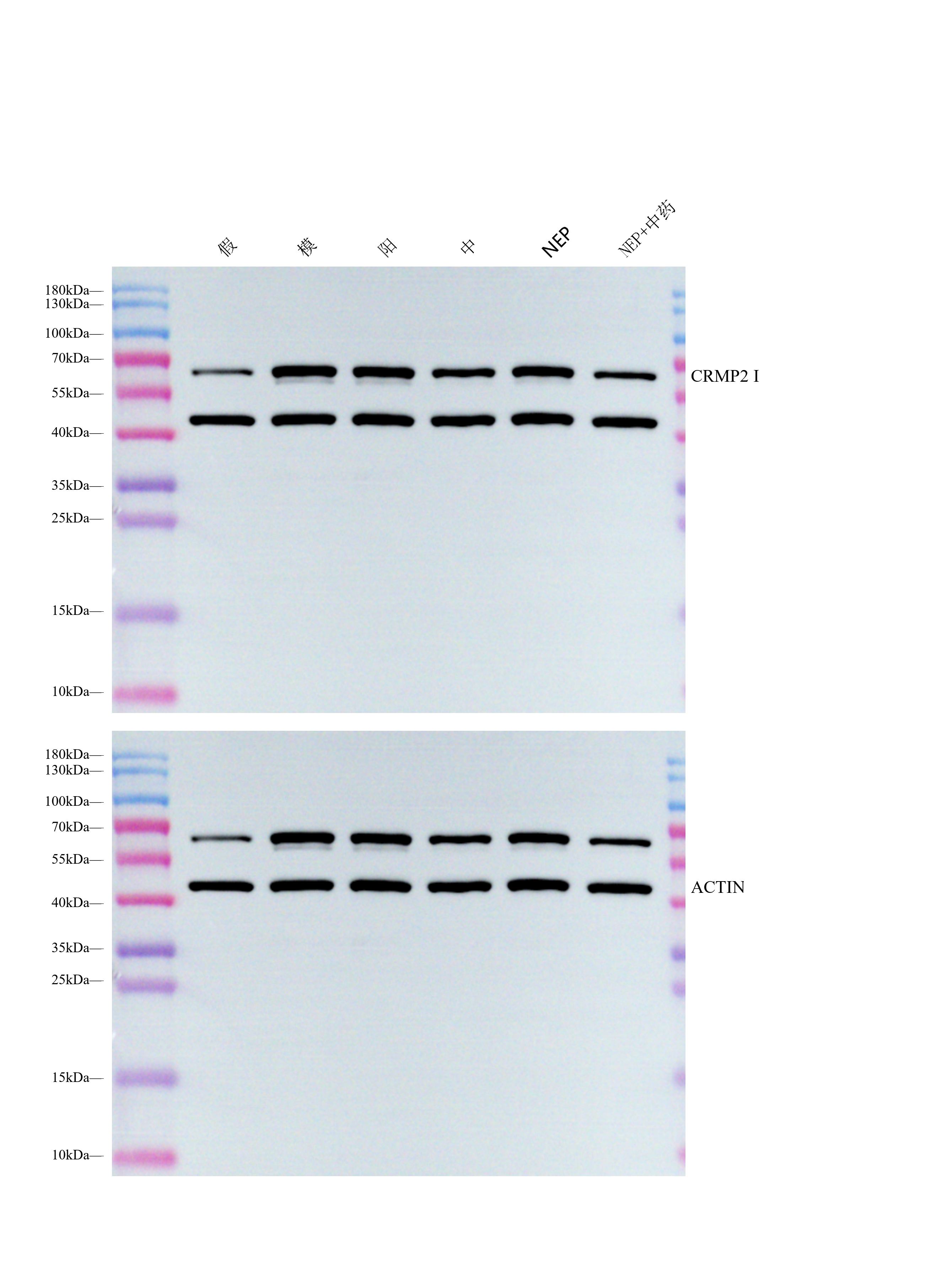

Supplement: Supplementary file 23 — Supplementary Information [file BRB3-16-e71170-s023.tif]

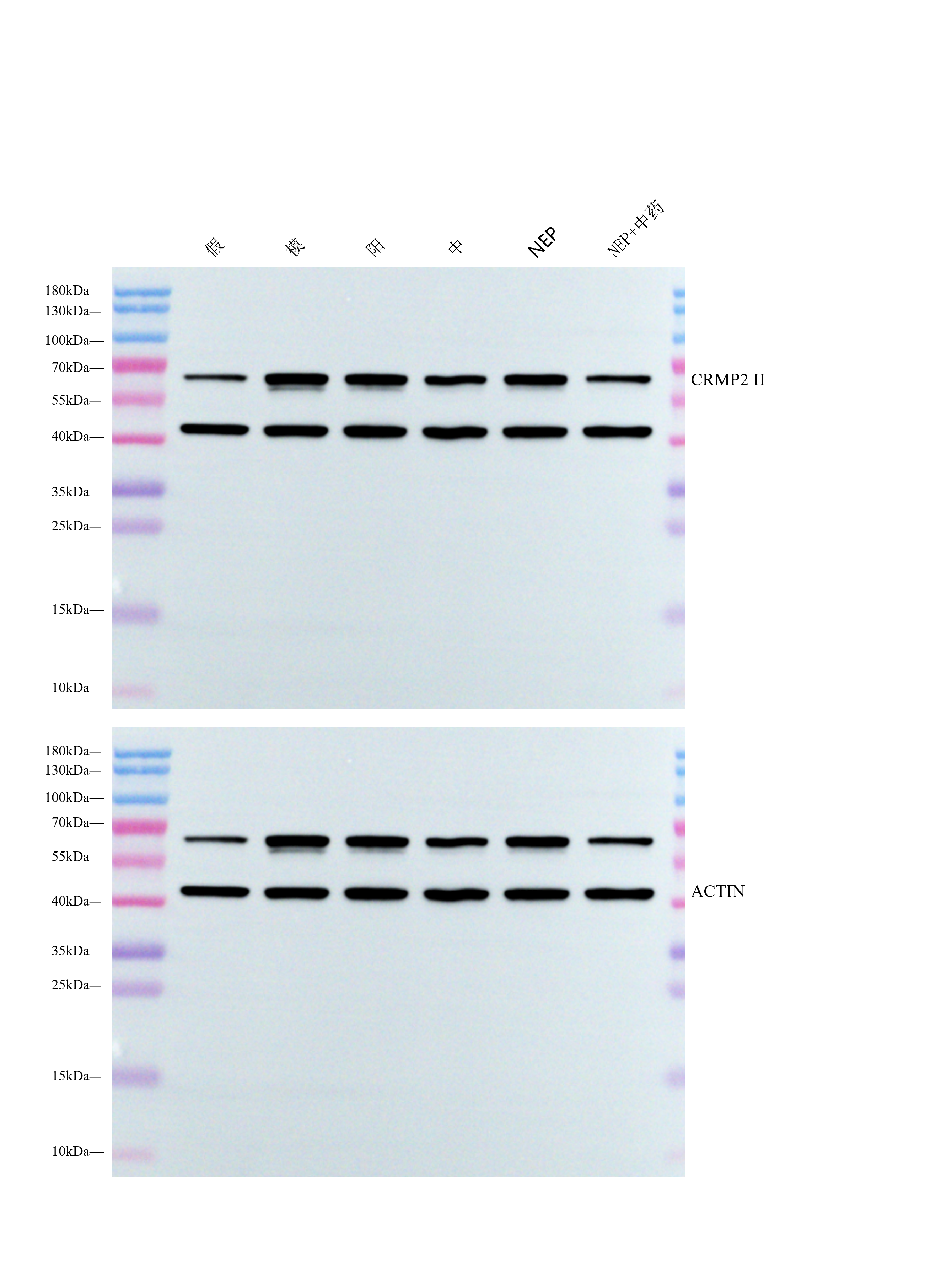

Supplement: Supplementary file 24 — Supplementary Information [file BRB3-16-e71170-s017.tif]

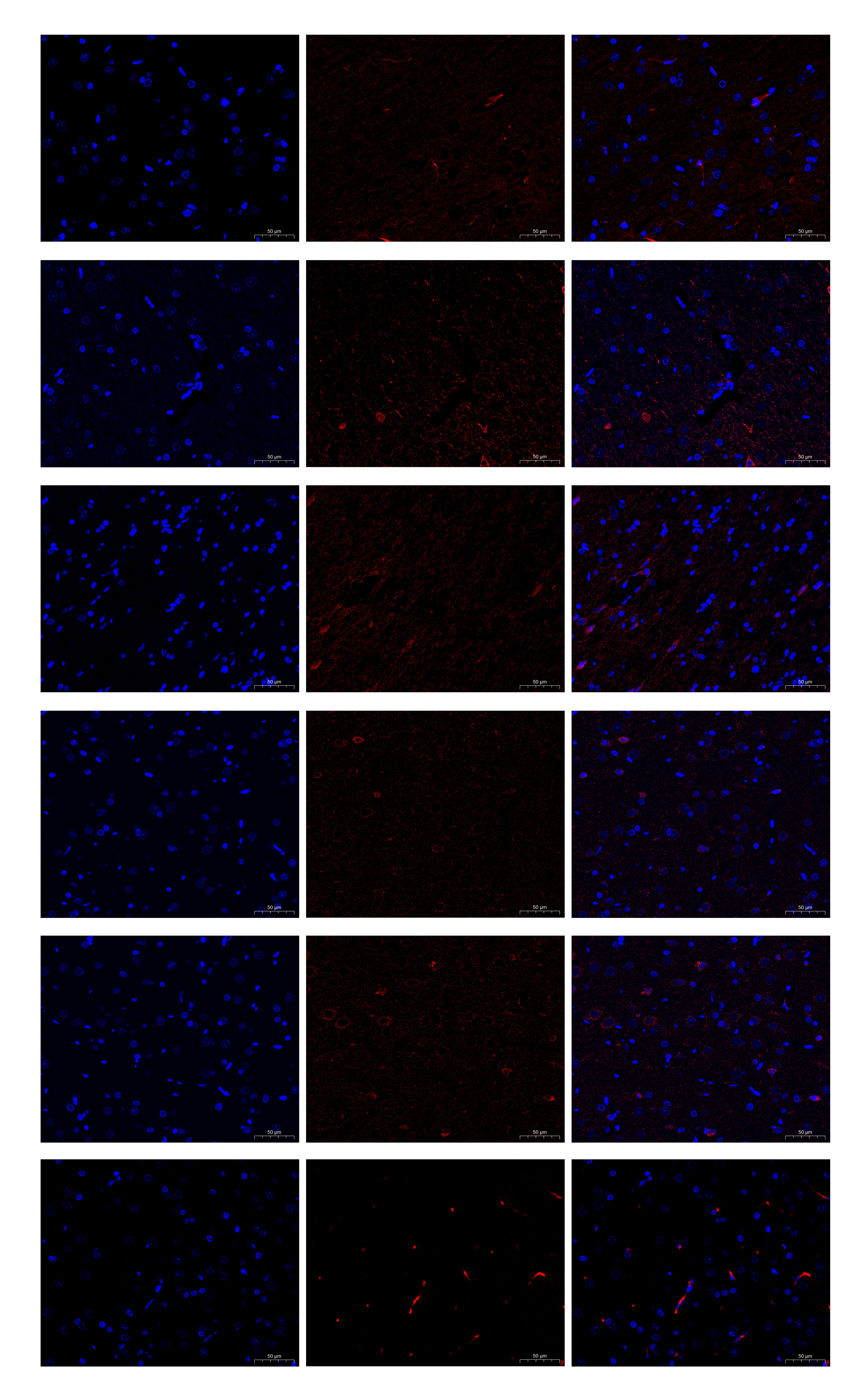

Supplement: Supplementary file 25 — Supplementary Information [file BRB3-16-e71170-s030.jpg]

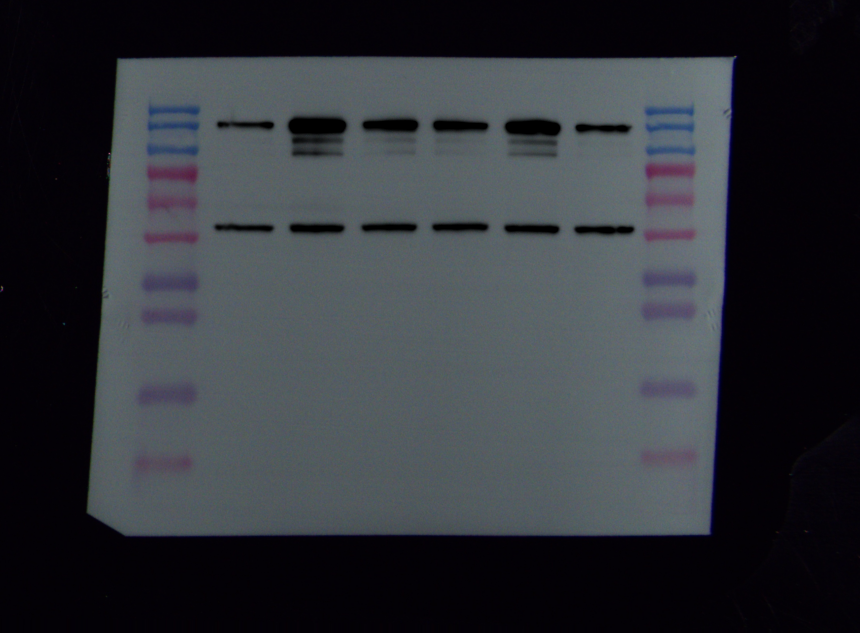

Supplement: Supplementary file 27 — Supplementary Information [file BRB3-16-e71170-s028.tiff]

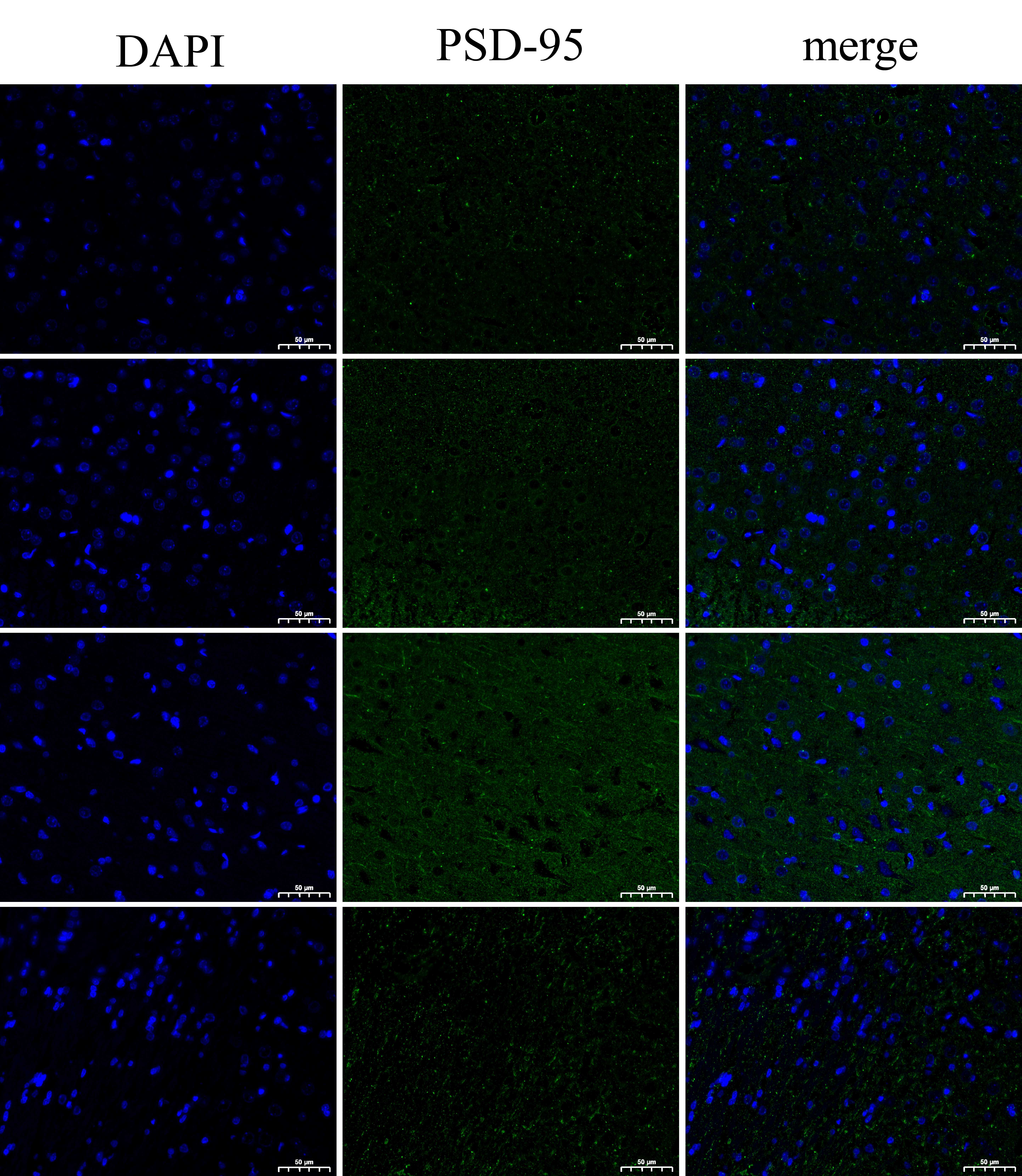

Supplement: Supplementary file 28 — Supplementary Information [file BRB3-16-e71170-s029.jpg]

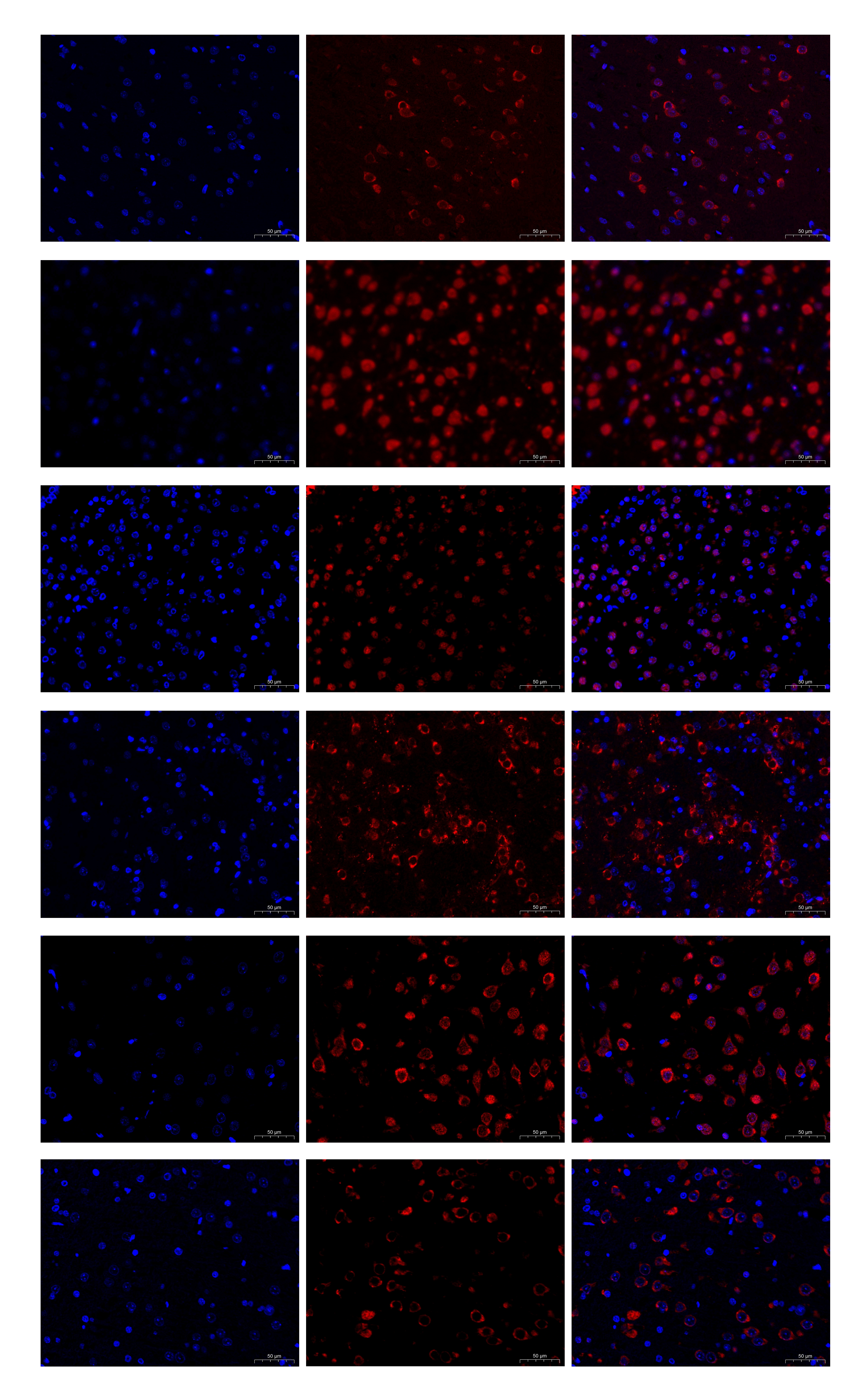

Supplement: Supplementary file 29 — Supplementary Information [file BRB3-16-e71170-s011.jpg]

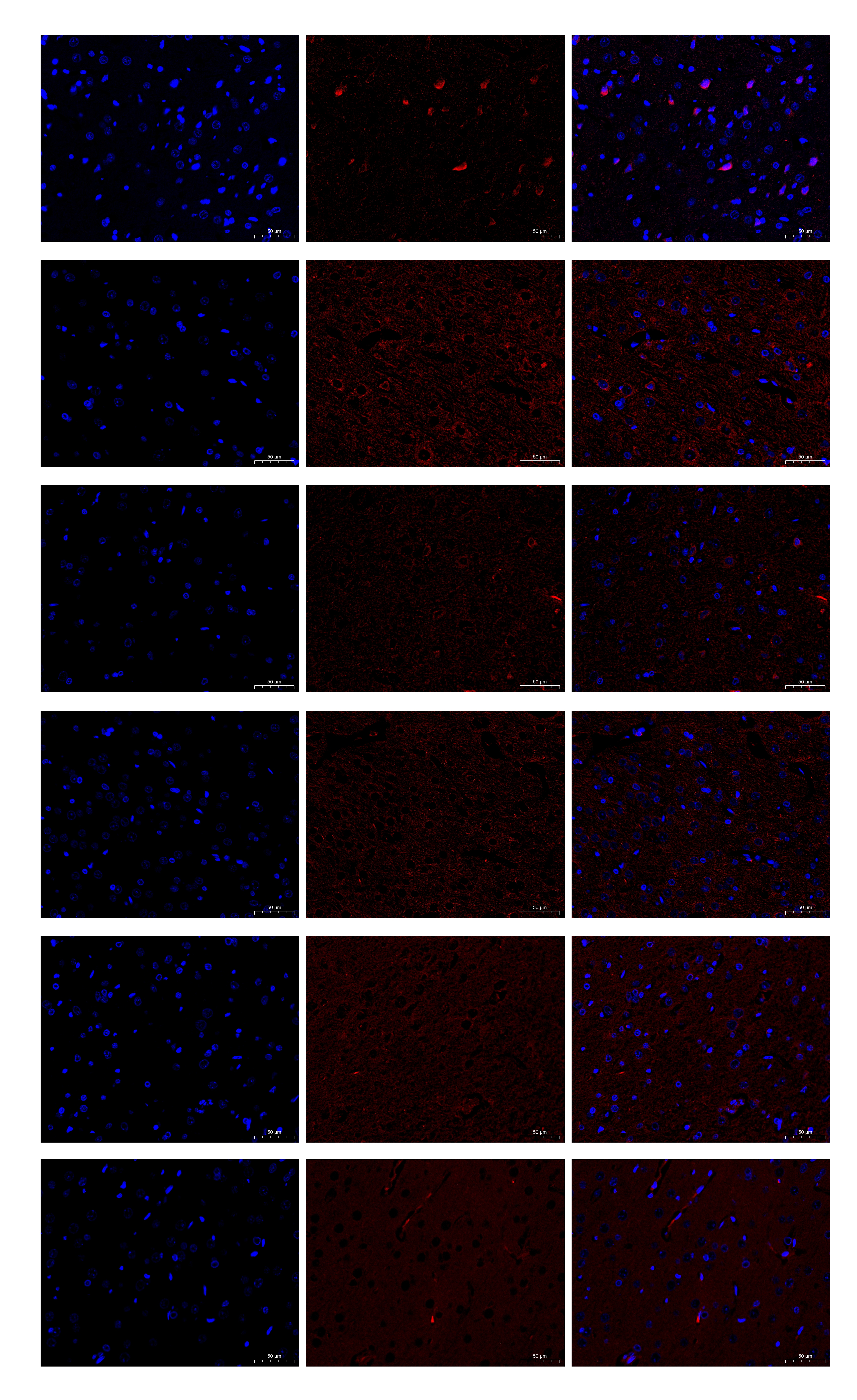

Supplement: Supplementary file 30 — Supplementary Information [file BRB3-16-e71170-s014.jpg]

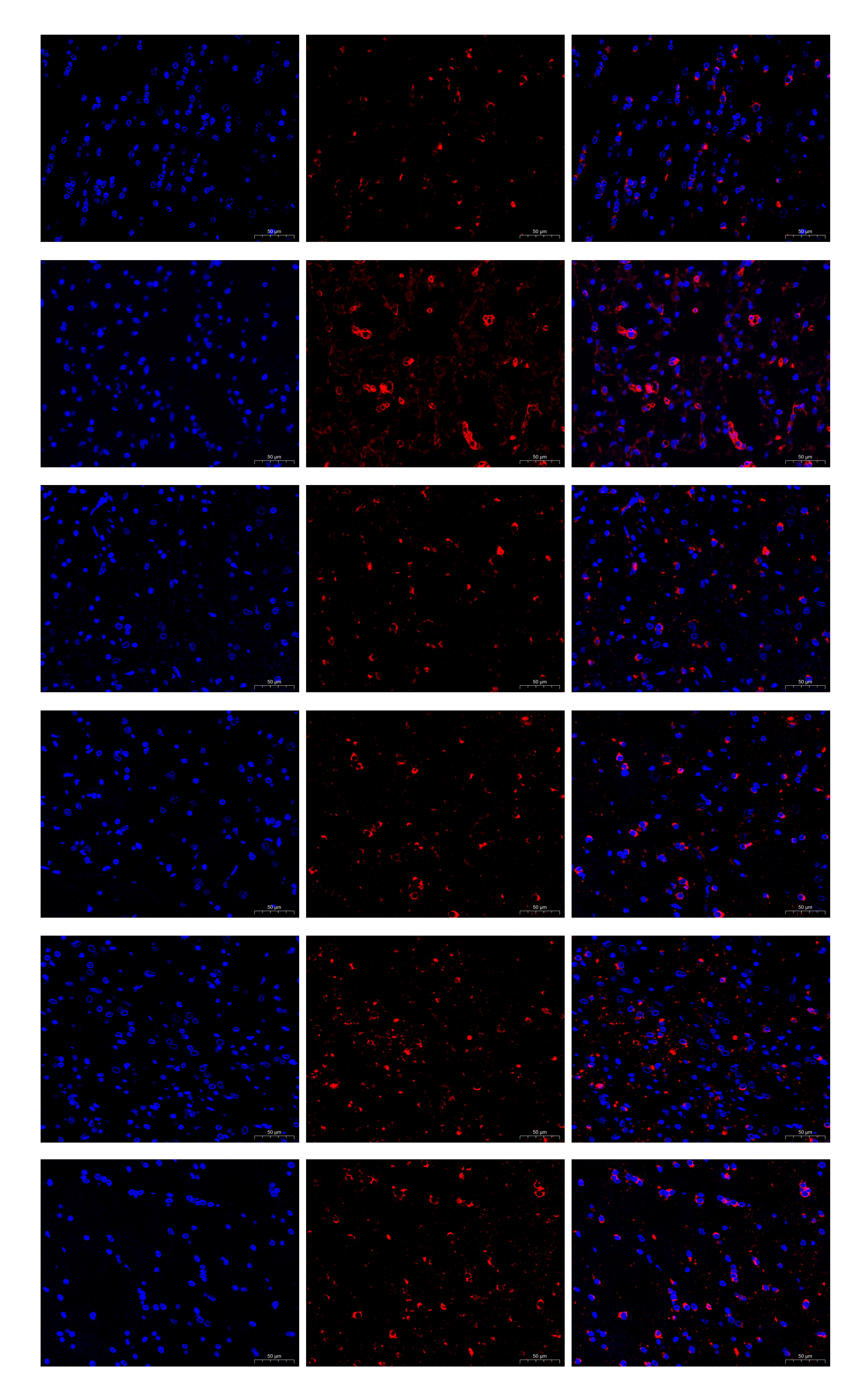

Supplement: Supplementary file 31 — Supplementary Information [file BRB3-16-e71170-s035.jpg]

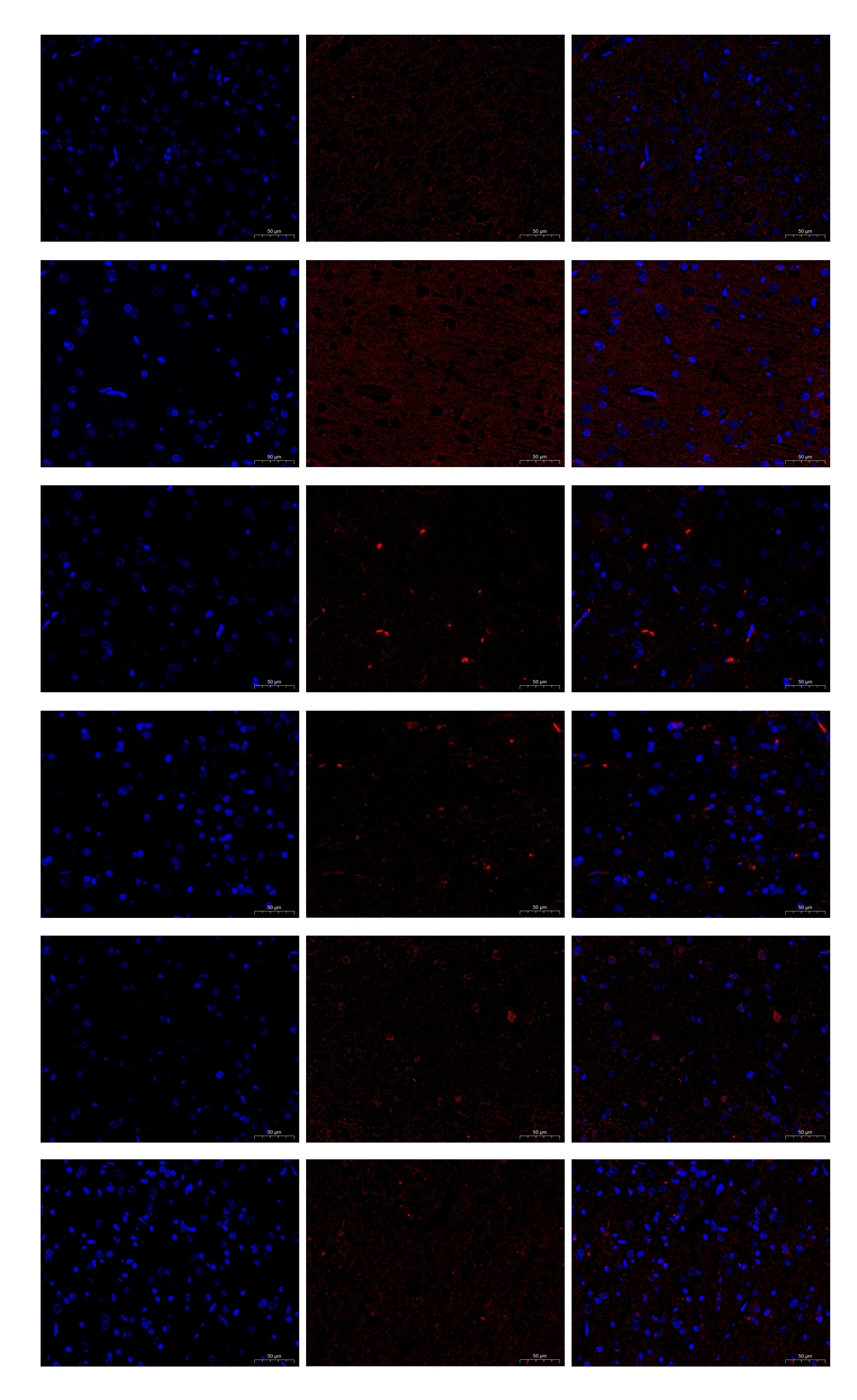

Supplement: Supplementary file 32 — Supplementary Information [file BRB3-16-e71170-s021.jpg]

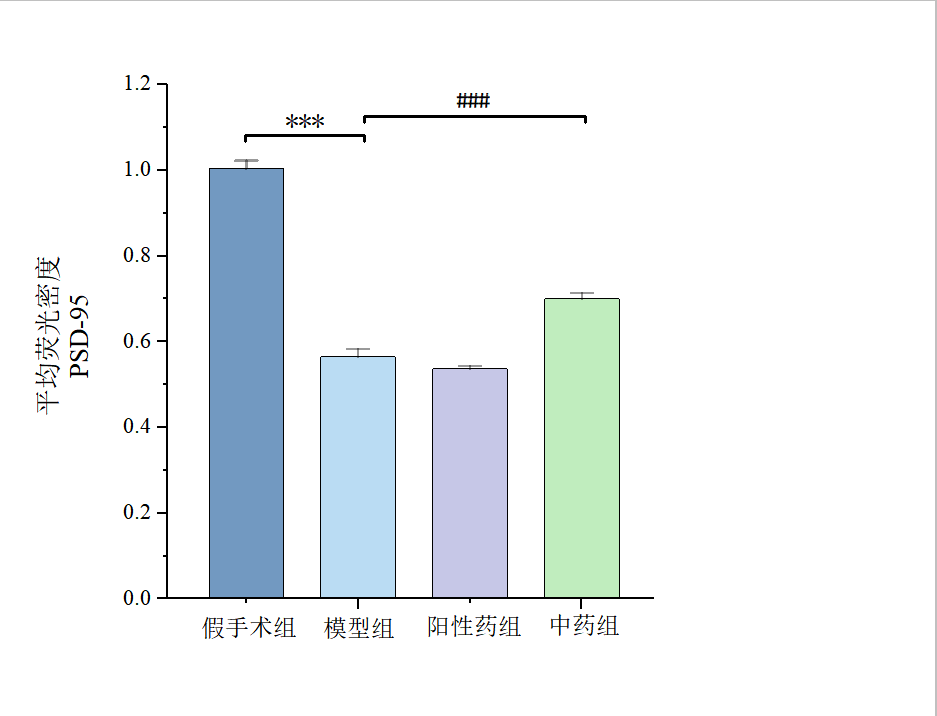

Supplement: Supplementary file 33 — Supplementary Information [file BRB3-16-e71170-s034.png]

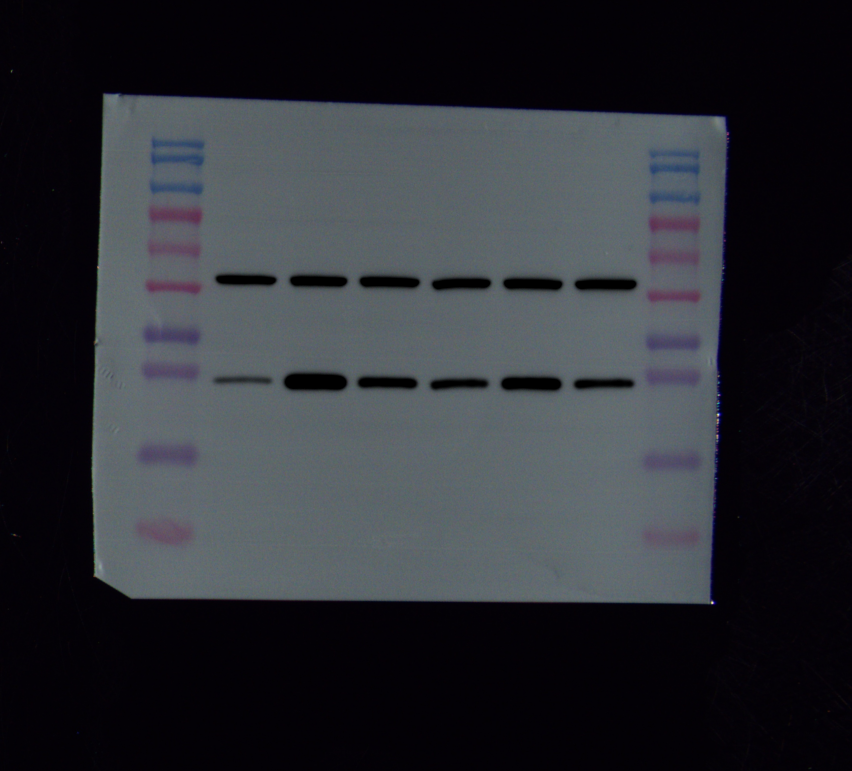

Supplement: Supplementary file 34 — Supplementary Information [file BRB3-16-e71170-s012.tiff]

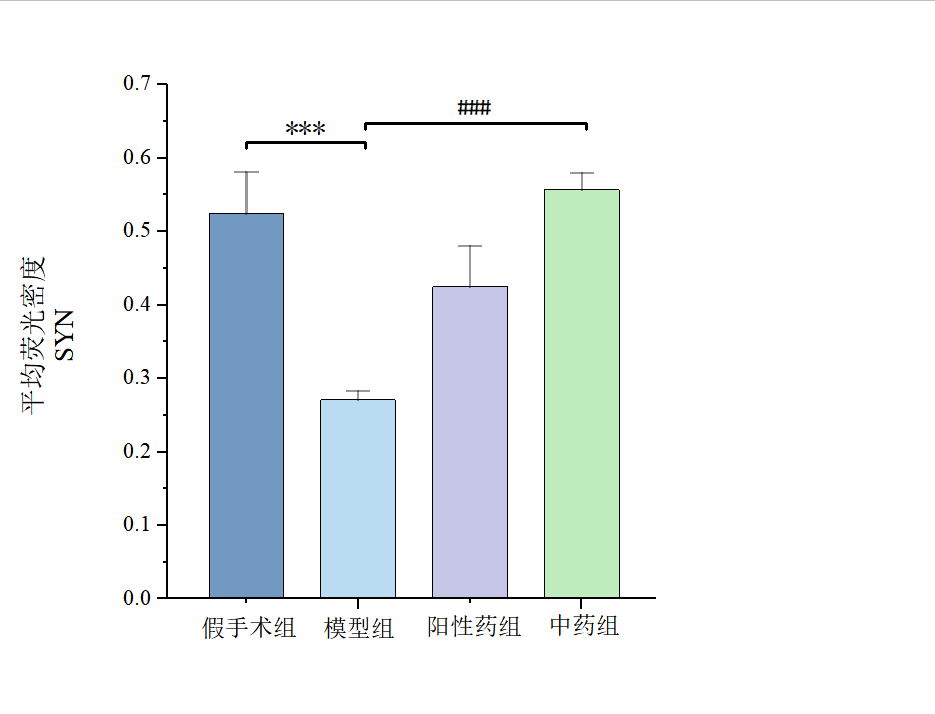

Supplement: Supplementary file 35 — Supplementary Information [file BRB3-16-e71170-s004.png]

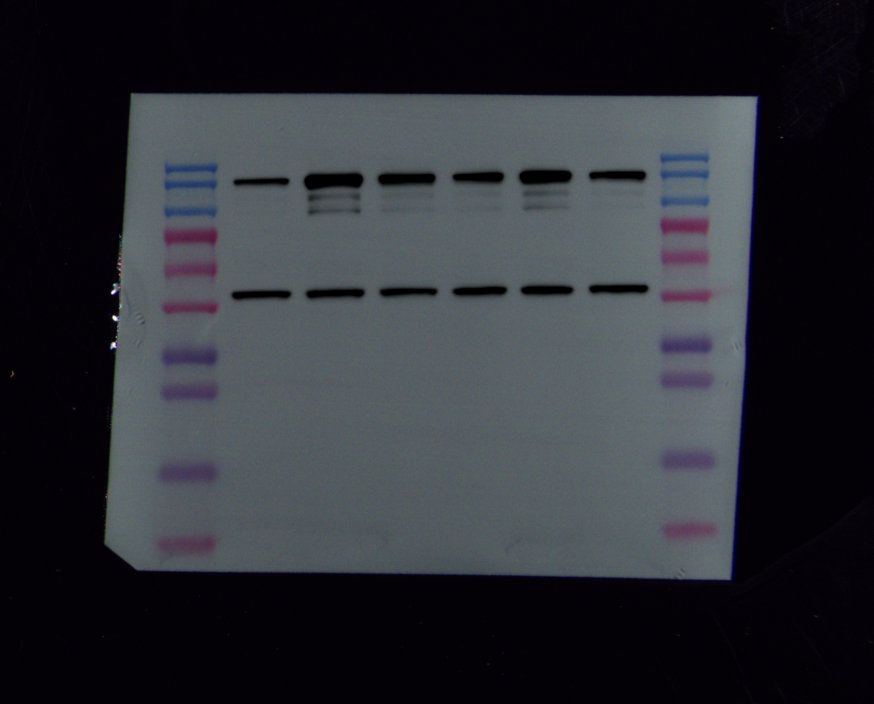

Supplement: Supplementary file 36 — Supplementary Information [file BRB3-16-e71170-s019.tiff]

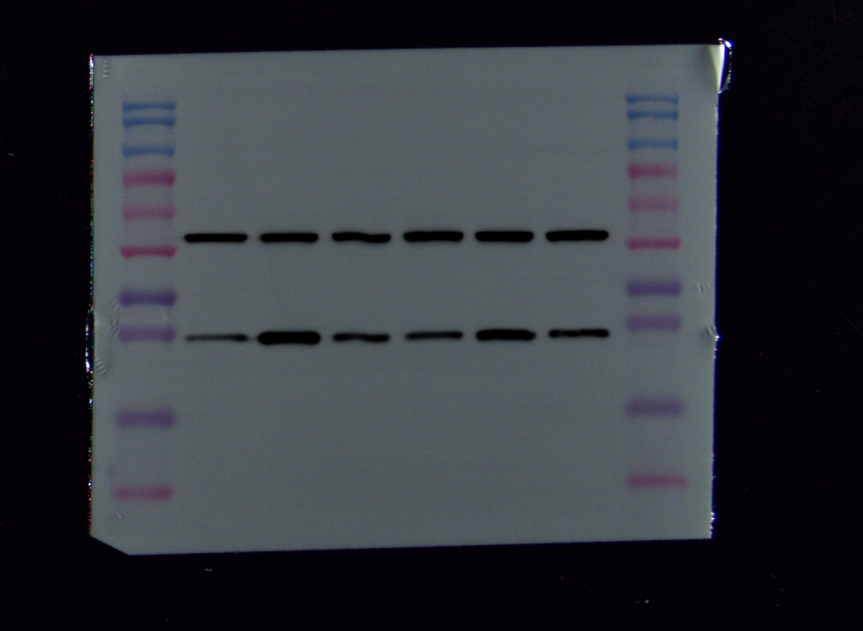

Supplement: Supplementary file 37 — Supplementary Information [file BRB3-16-e71170-s025.tiff]

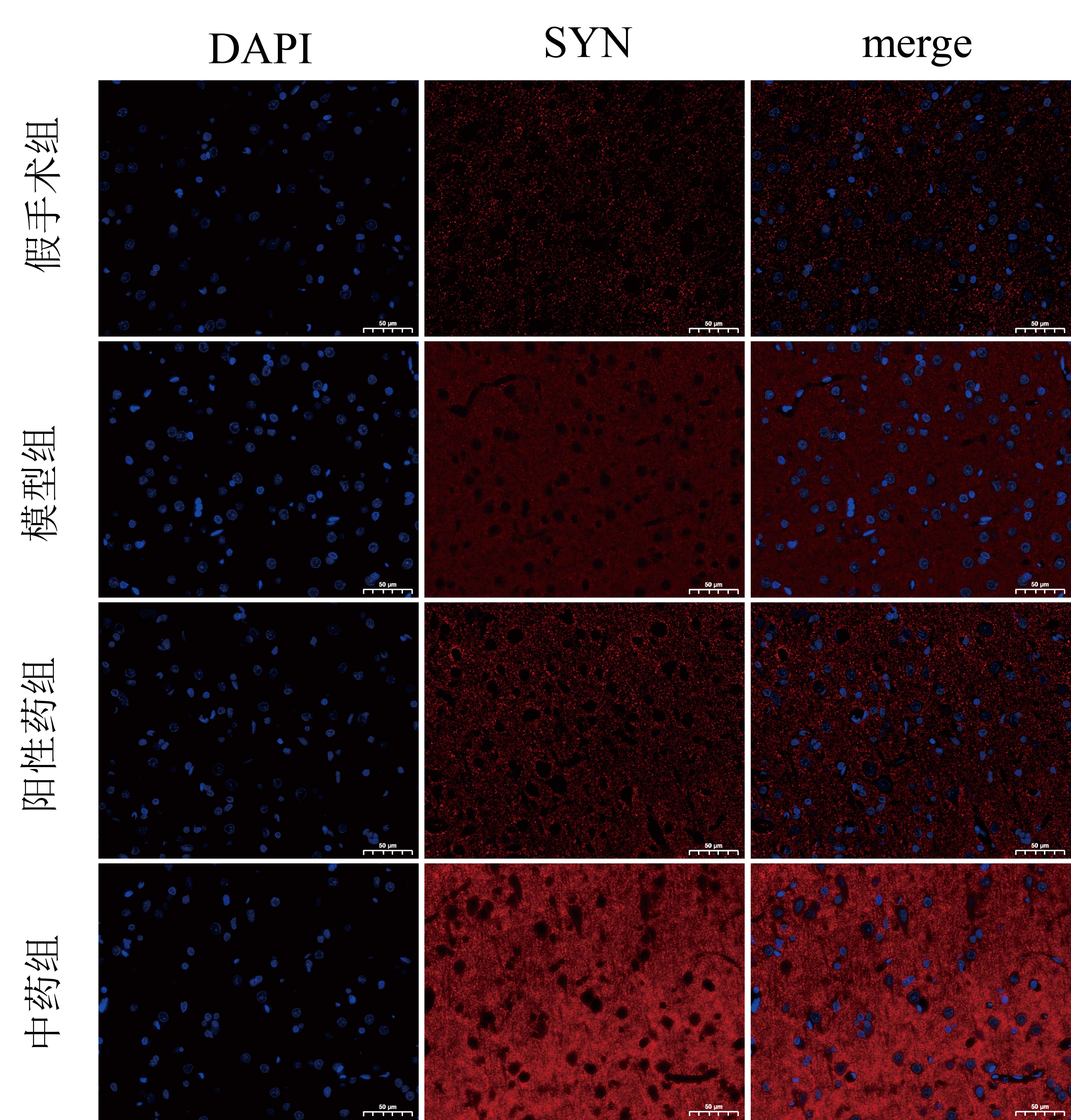

Supplement: Supplementary file 38 — Supplementary Information [file BRB3-16-e71170-s015.jpg]
