# Supplementary material for: Baishaoluoshi Decoction Mitigates Post‐Stroke Spasticity by Targeting Synaptic Plasticity Through the Nogo‐A/NgR Signaling Pathway
Source: Brain Behav. 2025 Dec 31;16(1):e71170. doi: 10.1002/brb3.71170 (PMC12755059; doi:10.1002/brb3.71170)
Supplement: Supplementary file 26 — Supplementary Information [file BRB3-16-e71170-s005.tif]

502 Bad Gateway


# 502 Bad Gateway
